# The Host Cell Factor Phosphatase‐2A Subunit PR130 Restricts Replication of Herpes Simplex Virus Type‐1

**DOI:** 10.1002/advs.202523697

**Published:** 2026-05-15

**Authors:** Johannes Jungwirth, Christoph F. Jacob, Alexandra Nguyen, Mandy Beyer, Andreas O. Mieland, Mario Dejung, Jia‐Xuan Chen, Walburgis Brenner, Christina Ehrhardt, Andreas Henke, Oliver H. Krämer

**Affiliations:** ^1^ Section of Experimental Virology Institute of Medical Microbiology Center for Molecular Biomedicine (CMB) Jena University Hospital Jena Germany; ^2^ Institute of Toxicology University Medical Center of the Johannes Gutenberg University Mainz Mainz Germany; ^3^ Institute of Molecular Biology gGmbH (IMB) Proteomics Core Facility Mainz Germany; ^4^ Department of Obstetrics and Gynecology University Medical Center of the Johannes Gutenberg University Mainz Mainz Germany

**Keywords:** ATM, CDK2, HSV‐1, lytic infection, p21, PP2A, PR130

## Abstract

Herpes simplex virus type‐1 (HSV‐1) affects over 60% of the human population and increasingly develops resistance to antiviral therapies. Efficient HSV‐1 replication requires host‐derived deoxynucleotide triphosphates and the manipulation of cellular DNA replication and repair. This work positions the protein phosphatase 2A (PP2A) regulatory subunit PR130 (PPP2R3A) as cellular factor that suppresses the replication of laboratory strains and clinical isolates of HSV‐1 in epithelial and neuronal cells. HSV‐1 infection in turn decreases PR130 levels. Global proteome and phosphoproteome profiling combined with functional assays demonstrate that PR130 modulates key regulators of the cell cycle and DNA repair. PR130 controls the expression and phosphorylation of the cyclin‐dependent kinase (CDK) inhibitor p21 (CDKN1A) at serine 130 (S130) which CDK2 catalyzes. The levels and activities of p21, which HSV‐1 infection attenuates, and CDK2 are decisive factors for HSV‐1 replication. Inhibition of the ubiquitin‐specific protease USP7 stabilizes the p53‐p21 axis and reduces HSV‐1 viral titers. Additionally, PR130 depletion enhances signaling of the DNA damage‐responsive checkpoint kinase ataxia‐telangiectasia mutated (ATM) upon HSV‐1 infection and creates a dependency of HSV‐1 replication on ATM activity. These findings uncover host‐intrinsic mechanisms regulating HSV‐1 replication and highlight PR130 as central hub regulator of HSV‐1 infection.

## Graphical abstract

Molecular, genetic, virological, and biochemical analysis in combination with global proteome and phosphoproteome profiling and functional assays were applied to study the role of PR130 in the context of HSV‐1 replication. The observations reveal that host‐intrinsic mechanisms regulate HSV‐1 replication and highlight PR130 as a susceptibility factor of viral replication as well as a central hub regulator upon HSV‐1infection.

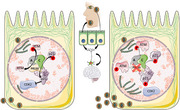

## 1 Introduction

The double‐stranded DNA virus herpes simplex virus type 1 (HSV‐1) belongs, together with HSV‐2 and the varicella‐zoster virus (VZV), to the subfamily of the *α‐Herpesvirinae* [1]. According to the World Health Organization (WHO), around 3.8 billion people (64.2%) under the age of 50 are estimated to be infected with HSV‐1 [2]. HSV‐1 infects epithelial cells of the human skin, mucosa, and the human eye tissues [3]. Symptoms of HSV‐1 infections include cold sores at the orolabial region and/or corneal keratitis [4]. In newborns or immunologically compromised individuals, HSV‐1 infection is often associated with severe systemic dissemination. This complication can result in encephalitis, meningitis, and blindness [5]. After primary infection, lifelong latency may be accompanied by periodical reactivation of viral replication, causing acute symptoms [6]. In the brain, reactivated HSV‐1 can provoke severe encephalitis. In addition, HSV‐1 might trigger the neuropathological sequela of Alzheimer disease [7].

Antiviral compounds shorten the healing time and lessen the severity of symptoms by targeting viral DNA replication. These include the nucleoside analogue acyclovir (ACV) and its derivatives as well as other nucleotide analogues like cidofovir [8]. However, HSV‐1 infections are not curable [8] and the excessive use of these few substances that target the viral DNA polymerase (POL), has led to the emergence of drug‐resistant strains [9]. The prevalence of viral ACV resistance in immunocompetent individuals is up to 0.7%, whereas in immunocompromised patients it is estimated to range between 2.1‐10.9% [10]. In 95% of the cases, resistance to ACV is mediated by mutations in the viral thymidine kinase (TK), and in the remaining cases, by mutations in the viral POL [11, 12]. To treat ACV‐resistant strains, helicase‐primase inhibitors such as amenamevir, and non‐nucleoside analogues to pyrophosphate like foscarnet, are used [13, 14]. To date, no vaccination against HSV‐1 is available for clinical use. Therefore, the development of new antiviral drugs against viral or cellular factors that promote HSV‐1 replication is a pressing need. To achieve this goal, more detailed insights into viral and host cell factors that control HSV‐1 replication are necessary.

Upon the entry of HSV‐1 into the cell and the transport of the viral DNA into the nucleus, the cellular RNA polymerase II transcribes viral proteins in a cascade‐dependent manner [15]. Viral immediate‐early (IE) proteins promote the transcription of early (E) genes, which are involved in viral DNA replication and the transcription of late (L) genes [16, 17]. As typical DNA virus, HSV‐1 exploits the cellular DNA synthesis machinery which is most active in the S phase of the cell cycle to replicate its genome [18, 19, 20]. Mammalian cells have a complex evolutionarily conserved program that ensures the error‐free replication of their DNA. DNA replication stress and DNA damage activate the checkpoint kinases ataxia telangiectasia mutated (ATM), ATM and RAD3‐related (ATR), DNA‐dependent protein kinase (DNA‐PK), checkpoint kinases‐1/‐2 (CHK1/CHK2), and WEE1 [21, 22]. These proteins slow down cell cycle progression by various mechanisms, including the suppression of cyclin‐dependent kinases (CDKs). CDKs generate catalytically active complexes that interact with the cell cycle‐dependently expressed cyclins. The balance between the CDKs CDK1/CDK2/CDK4/CDK6 and the cyclins A/B/D/E fine‐tunes cell cycle entry and progression as well as proper mitosis [21, 22]. HSV‐1 genomes, when entering the nucleus as linear double‐strand DNA molecules, resemble cellular DNA with single‐ and double‐stranded DNA breaks [23]. These consequently activate the checkpoint kinases ATM, ATR, and DNA‐PK, and their target proteins CHK1, CHK2, and WEE1 by phosphorylation [24, 25, 26, 27, 28, 29, 30, 31, 32, 33, 34]. HSV‐1 has evolved mechanisms that exploit and manipulate these signaling cascades. The infected cell protein 0 (ICP0) of HSV‐1 promotes proteasomal degradation of DNA‐PK to disable DNA repair by non‐homologous end‐joining (NHEJ) which can limit HSV‐1 replication [23, 24, 25]. Consistent with this, HSV‐1 activates the Fanconi anemia DNA repair pathway which disfavors NHEJ [35]. Another NHEJ protein, PAXX promotes HSV‐1 genome replication efficiency [36], and RNAi‐mediated knockdown of the NHEJ‐associated DNA‐ligase IV and its co‐factor XRCC4 reduces HSV‐1 production [37]. Such contradictory results may be explained by delayed cell cycle progression upon lost DNA damage resolution [38]. DNA‐PK has further roles for the HSV‐1 infection cycle. It phosphorylates cellular and viral proteins to repress IE gene expression that initiates the HSV‐1 replication program and DNA‐PK promotes heterochromatinization for a consequent silencing of HSV‐1 transcription [39, 40, 41]. The situation appears also complex for ATM. ATM activation enhances HSV‐1 replication compartment formation and productive infection [27, 42, 43, 44] and ATM activation opposes the DNA‐PK driven expression of antiviral interferon‐β (IFNβ) [45]. ATR exerts pro‐replicative functions for HSV‐1. Inhibition of ATR by its specific inhibitor VE‐821 and siRNA against ATR reduces HSV‐1 replication. ATR enhances HSV‐1 replication by promoting the resolution of replication‐associated DNA structures and by preventing DNA replication fork collapse on viral DNA [27, 41, 46]. ATR‐activated CHK1 seems to act proviral and ATM‐activated CHK2 has relevance for HSV‐1 replication in some cell types [26, 41, 47]. These datasets illustrate that significantly more work on the impact of checkpoint kinases is required to understand their functions in various cell types and to prospectively exploit them therapeutically against HSV‐1.

Protein phosphatase 2A (PP2A) is a major enzyme for the dephosphorylation of serine and threonine residues. PP2A complexes consist of scaffolding A subunits, regulatory B subunits ensuring substrate selectivity, and catalytic C subunits. Trimeric PP2A complexes regulate key physiological processes such as cell cycle progression (e.g., WEE1, CDC25, pRB), apoptosis (e.g., BCL2, BAD, FOXO), and DNA damage repair (e.g., p53, ATR, ATM, CHK1, CHK2). There are multiple PP2A B‐type subunits. For example, PR130 (B″α1) and its splice variant PR72 (B″α2) are two of at least 24 B subunits of PP2A [48]. Unbiased global assays, such as proteomic and phosphoproteomic analyses, are necessary to comprehensively understand through which molecular mechanisms PP2A family members may modulate viral infection and spread.

This work demonstrates for the first time that the PP2A‐PR130 complex has an impact on HSV‐1 replication through ATM and the p21‐CDK2 axis.

## 2 Results

### 2.1 The PP2A B‐type Subunit PR130 Regulates HSV‐1 Replication in Epithelial Cells

**FIGURE 1:**
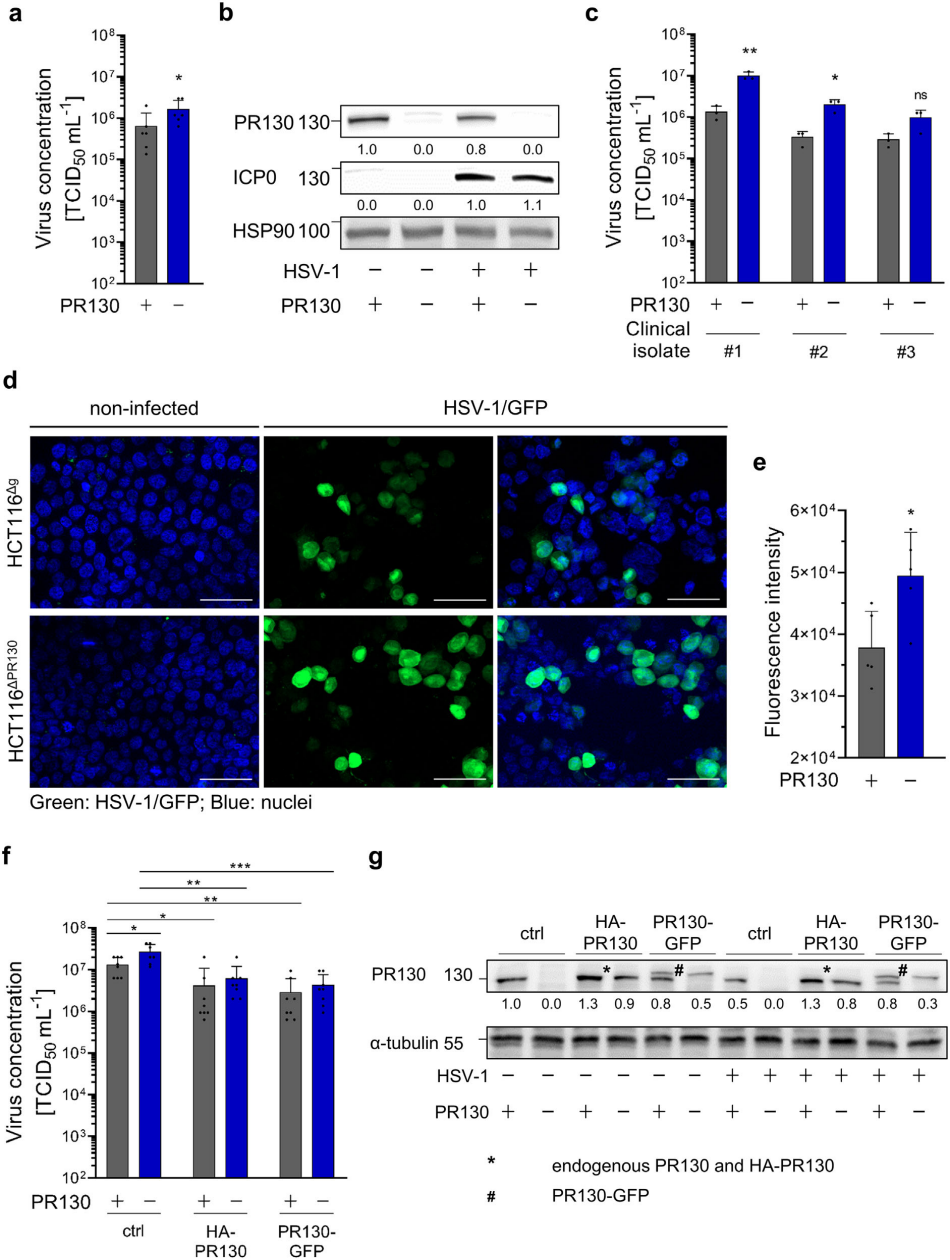
The PP2A B‐type Subunit PR130 Regulates HSV‐1 Replication in Epithelial Cells. (a,b,c) HCT116^Δg^ and HCT116^ΔPR130^ cells were infected with (a,b) HSV‐1/GFP using a MOI of 1 or (c) 3 different clinical isolates of HSV‐1 (#1, #2, and #3) using a MOI of 1. (a,c) Virus concentrations were determined in supernatants 24 h p.i. Results of virus titrations are means (+SD) of TCID_50_ mL^−1^ values of 3 independent experiments, including 2 biological replicates (TCID_50_ mL^−1^ equates to infectious virus concentration). (b) Immunoblots were performed 24 h p.i. for PR130, HSV‐1 ICP0, and HSP90 as control for equal sample loading. Numbers next to protein names indicate the molecular weight of the protein marker in kDa. Numbers below represent the means of 3 densitometric analyses relative to the loading control and normalized to the non‐infected or infected control cells (Figure S7a). (d) HCT116^Δg^ and HCT116^ΔPR130^ cells were infected with HSV‐1/GFP using a MOI of 1. After 24 h p.i., cells were fixed and nuclei were stained with Hoechst 33342. Samples were evaluated by fluorescence microscopy. Scale bars represent 50 µm. Representative images of 3 independent experiments are displayed. (e) The z‐stacks of (d) were maximum intensity projected and mean fluorescence intensities per image were determined. Five images were analyzed using FIJI's inbuilt measure function and depicted as means (+SD). (f,g) HCT116^Δg^ and HCT116^ΔPR130^ cells were transfected with 3 µg plasmid DNA and infected with HSV‐1/KOS using a MOI of 1. After a medium exchange, cells were further incubated in plasmid‐containing medium. (f) Virus concentrations were determined in supernatants 24 h p.i. Results of virus titrations are means (+SD) of TCID_50_ mL^−1^ values of 4 independent experiments, including 2 biological replicates. (g) Immunoblots were performed 24 h p.i. for PR130, HSV‐1 ICP0, and α‐tubulin as control for equal sample loading. Numbers next to protein names indicate the molecular weight of the protein marker in kDa. Numbers below represent the means of 3 densitometric analyses relative to the loading control and normalized to the non‐infected or infected control cells (Figure S7b). (a,c,e,f) Statistical significance was analyzed by unpaired two‐tailed *t*‐tests against the control cells (ns, not significant; *, *p* ≤ 0.05; **, *p* ≤ 0.01; ***, *p* ≤ 0.001).

Since PR130 controls cellular DNA replication stress responses [49], we hypothesized that PR130 also affected the replication of DNA viruses, including HSV‐1. To test this idea, we infected CRISPR‐Cas9‐based PR130‐depleted epithelial colorectal HCT116 cells (HCT116^ΔPR130^) and corresponding control cells (HCT116^Δg^) with HSV‐1/GFP. If not mentioned otherwise, we used this HSV‐1 strain for infection experiments. Compared to HCT116^Δg^ cells, HCT116^ΔPR130^ cells produced significantly more HSV‐1 24 h post infection (p.i.) (Figure 1a). Immunoblot analysis confirmed PR130 expression in HCT116^Δg^ cells and its absence in HCT116^ΔPR130^ cells. This analysis additionally revealed that the infection with HSV‐1 decreased the levels of PR130 in HCT116^Δg^ cells (Figure 1b). HSV‐1 IE genes are transcribed first after viral entry and without the need for de novo protein synthesis [16, 17]. We detected the viral IE gene product ICP0 as readout for the onset of early viral infection steps. An equal expression of ICP0 in HCT116^Δg^ and HCT116^ΔPR130^ cells (Figure 1b) disfavors differences in viral entry and viral IE gene expression as explanation for the higher HSV‐1 production by HCT116^ΔPR130^ cells (Figure 1a).

To exclude that higher viral titers in HCT116^ΔPR130^ cells than in HCT116^Δg^ cells were caused by different cytotoxic effects of HSV‐1 in these systems, we conducted the WST‐1 metabolic activity test, microscopic analyses, and immunoblots for the cleavage of PARP1 being a marker for apoptotic and necrotic cell death (89 kDa and 55 kda cleavage products, respectively). These virus‐induced cytopathic effects did not differ between all samples tested and there was no massive cytotoxicity at the time points of our analyses (Figure S1a–c).

Laboratory‐adapted HSV‐1 strains may not reflect the genetic diversity of viruses that circulate in human populations [50]. To study our above results for HSV‐1, we infected HCT116^ΔPR130^ as well as HCT116^Δg^ cells with the clinical isolates #1 (1076/98), #2 (1123/99), and #3 (598/02). Like the laboratory HSV‐1 strain, these clinical isolates reached higher titers in cells lacking PR130 than in HCT116^Δg^ cells (Figure 1c).

Consistent with this data, immunofluorescence microscopy showed increased replication activity of HSV‐1 in the absence of PR130 (Figure 1d). This observation is supported by quantitative measurements of GFP mean fluorescence intensity (Figure 1e). Since GFP is encoded from a constitutively active promoter on the HSV‐1 genome, increased GFP signals are indicative of increased viral load, i.e., increased virus replication.

To ensure that the variable production rates of infectious HSV‐1 in HCT116^Δg^ and HCT116^ΔPR130^ cells stem from the variable expression of PR130 and to define if the reduction of PR130 by HSV‐1 is functionally relevant, we reconstituted HCT116^ΔPR130^ cells with PR130. We transfected such cells with plasmids encoding HA‐PR130 [49] and PR130‐GFP. Congruent with the results that we collected through a genetic elimination of PR130 by CRISPR‐Cas9 and a resulting rise in HSV‐1 production (Figure 1a–e), an overexpression of both tagged variants of PR130 attenuated HSV‐1 concentrations (Figure 1f,g). That we also see reduced HSV‐1 production in HCT116^Δg^ cells overexpressing PR130 (Figure 1f,g) can be explained by higher levels of PR130 at the start and after a 24 h infection with HSV‐1.

These data illustrate that PR130 is a newly recognized suppressor of the replication of laboratory HSV‐1 strains and of fresh clinical HSV‐1 isolates and that HSV‐1 attenuates PR130 to increase cellular HSV‐1 production.

### 2.2 PR130 Suppresses Early HSV‐1 Replication Steps in Epithelial Cells

**FIGURE 2:**
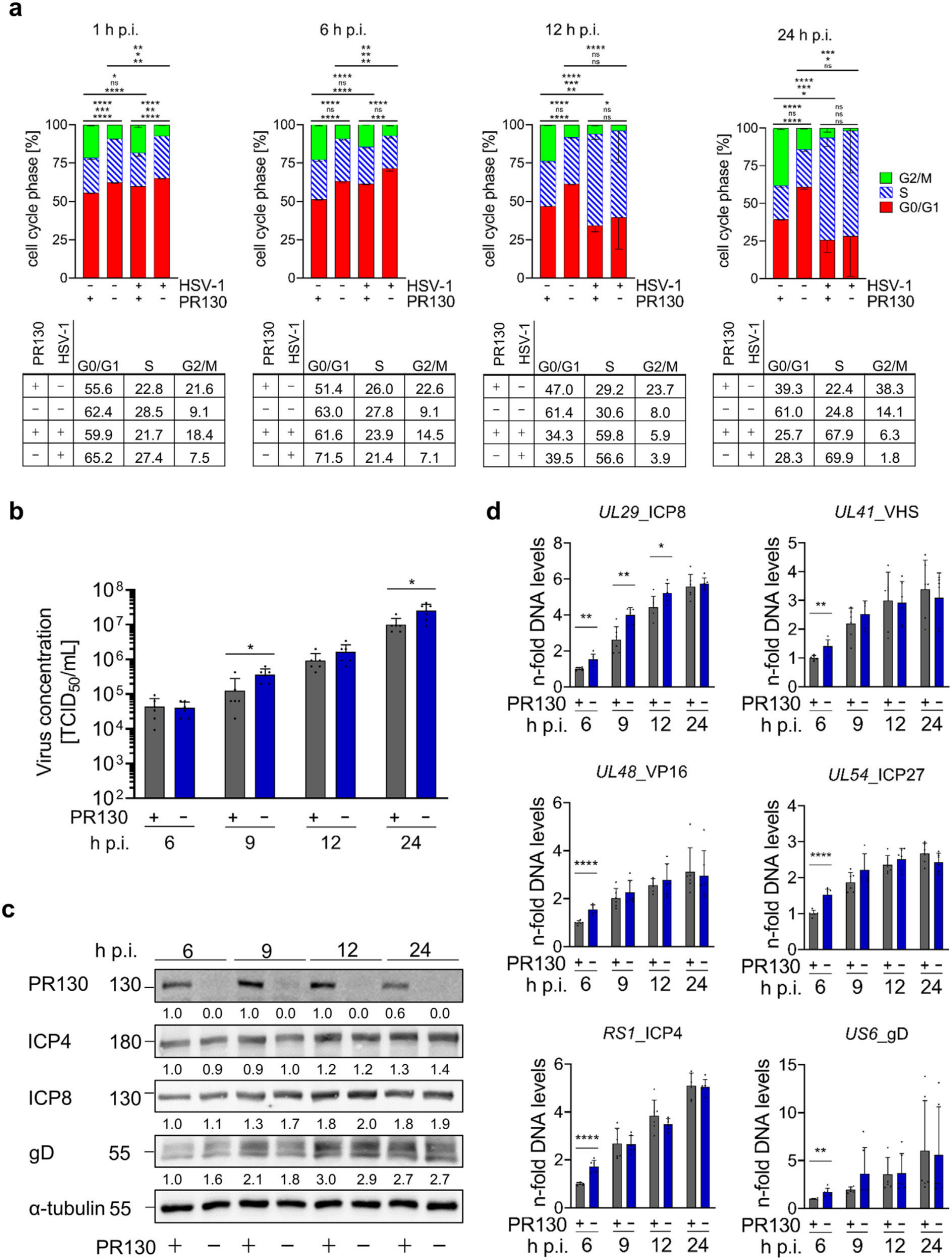
PR130 Suppresses Early HSV‐1 Replication in Epithelial Cells. HCT116^Δg^ and HCT116^ΔPR130^ cells were infected with (a) a MOI of 2 of HSV‐1/KOS (HSV‐1/GFP was avoided as GFP and PI give overlapping light signals) or (b‐d) a MOI of 1 of HSV‐1/GFP. After medium change, the cells were further incubated for up to 24 h p.i. (a) Cellular DNA contents were determined at the indicated times by flow cytometry. Processed cells were labelled with PI (10 µg/mL) to calculate different cell cycle phases. The data were acquired as means (‐SD) of 3 independent experiments, including 2 biological replicates, and were obtained using ModFit LT 6.0 software. Statistical significance was analyzed by unpaired two‐tailed *t*‐test and the results are shown (from top to bottom) for G2/M, S and G0/G1 phases (ns, not significant; *, *p* ≤ 0.05; **, *p* ≤ 0.01; ***, *p* ≤ 0.001; ****, *p* ≤ 0.0001). (b–d) At the indicated timepoints virus concentrations were determined in supernatants, cells were lysed for immunoblotting and DNA was isolated. (b) Results of virus titrations are means (+SD) of TCID_50_ mL^−1^ values of 3 independent experiments, including 2 biological replicates. (c) Immunoblots were performed for PR130, HSV‐1 ICP4, HSV‐1 ICP8, HSV‐1 gD, and α‐tubulin as a control for equal sample loading. Numbers next to protein names indicate the molecular weight of the protein marker in kDa. Numbers below represent the means of 3 densitometric analyses relative to the loading control and normalized to the 6 h p.i. control cells (Figure S7c). (d) Viral DNA levels were investigated by real time PCR. Results are means (+SD) of 2^−ΔΔC(T)^ values of the 6 h p.i. HCT116^Δg^ of 3 independent experiments, including 2 biological replicates. (b,d) Statistical significance was analyzed by unpaired two‐tailed *t*‐test (*, *p* ≤ 0.05; **, *p* ≤ 0.01; ****, *p* ≤ 0.0001).

We examined whether the differences in HSV‐1 replication in HCT116^Δg^ and HCT116^ΔPR130^ cells are linked to altered cell cycle progression. We infected the cells with the HSV‐1/KOS strain and collected them at different times p.i. (1, 6, 12, and 24 h). Samples were then stained with propidium iodide (PI) and processed by flow cytometry. We used the ModFit LT 6.0 software to evaluate cell cycle distributions and the percentages of cells in G0/G1, S, and G2/M phases. We noted higher numbers of G0/G1 and S phase cells in non‐infected HCT116^ΔPR130^ cells than in HCT116^Δg^ cells (Figure 2a). Twelve hours p.i., the infected cells accumulated in S phase. At 24 h p.i. there was an increase in the S phase populations; by 45% to 67% in infected HCT116^Δg^ cell cultures and by 45% to 69% in infected HCT116^ΔPR130^ cell cultures compared to non‐infected cultures (Figure 2a). These observations rule out gross cell cycle alterations as reason for the divergent HSV‐1 replication levels in HCT116^ΔPR130^ and HCT116^Δg^ cells.

To analyze through which mechanisms the PR130 status of cells alters HSV‐1 replication and viral protein expression, we performed immunoblots and real‐time PCR‐based quantifications of viral DNA at different timepoints within the viral replication cycle. The measurement of released infectious particles by TCID_50_ showed significantly higher HSV‐1 concentrations in HCT116^ΔPR130^ cell cultures than in HCT116^Δg^ cells at 9 and 24 h p.i. (Figure 2b). Analyzing earlier time points, we noted high HSV‐1 levels 1 h p.i., which stem from free HSV‐1 that was not taken up by the cells. This was followed by an eclipse phase between 3 and 6 h. That these processes occurred equally in HCT116^Δg^ and HCT116^ΔPR130^ cells corroborates that the uptake of the viral particles was not different in both cell systems (Figure 2b; Figure S1d).

Immunoblot analyses of cells that were infected for 6–24 h showed a trend for higher levels of the HSV‐1 IE protein ICP4, the E protein ICP8, and the L protein gD in HCT116^ΔPR130^ cells than in HCT116^Δg^ cells (Figure 2c). Such data is overlaid by higher TCID_50_ values for infected HCT116^ΔPR130^ cells, i.e., a higher release of infectious viruses containing these proteins (Figure 2b). Moreover, there is a time‐dependent decrease of PR130 in HCT116^Δg^ cells upon infection with HSV‐1 (Figure 2c).

To corroborate these datasets, we determined viral DNA levels directly by real time PCR. There were significantly higher levels of the HSV‐1 genes *UL29, UL41, UL48, UL54, RS1, and US6* in HCT116^ΔPR130^ cells than in HCT116^Δg^ cells at 6 h p.i. These differences disappeared at later timepoints (Figure 2d) which correlated with virus production and a reduction of PR130 in HCT116^Δg^ cells (Figure 2c,d).

Next, we assessed if the effect of PR130 on HSV‐1 replication can be extrapolated to other epithelial cell models. We engineered pancreatic MIA PaCa‐2 cells with a CRISPR‐Cas9‐based depletion of PR130. These proliferate like wild‐type MIA PaCa‐2 cells up to 48 h but show a delayed long‐term growth (Figure S2a). Upon 24 h p.i. with HSV‐1, we detected higher viral titers in MIA PaCa‐2^ΔPR130^ cells than in MIA PaCa‐2^Δg^ cells (Figure S2b). ICP0 was expressed equally in MIA PaCa‐2^Δg^ and MIA PaCa‐2^ΔPR130^ cells (Figure S2c).

We carried out additional analyses with cell systems carrying endogenous variations in the expression of PR130. We made use of our recent finding that subsets of murine pancreatic ductal adenocarcinoma (PDAC) short‐term cell cultures have significantly different PR130 mRNA and protein levels [51, 52]. As in the other cell systems that we analyzed, increased concentrations of HSV‐1 were produced by cells with reduced PR130 expression (Figure S2d,e).

These findings suggest that the levels of the host cell protein PR130 regulate HSV‐1 replication in various epithelial cell types.

### 2.3 PR130 Antagonizes HSV‐1 Replication in Nerve and Retinal Epithelial Cells

HSV‐1 establishes primary infections in cells of human lips or eyes, followed by a latent state in neurons [7]. Having found that PR130 is a negative regulator of HSV‐1 replication, we analyzed the expression of PR130 in cells of human tissues that HSV‐1 infects. The Human Proteome Atlas indicates the presence of PR130 in skin, eye, and brain tissues (Figure S3a–c), which suggests that it can affect the natural course of HSV‐1 infection.

**FIGURE 3:**
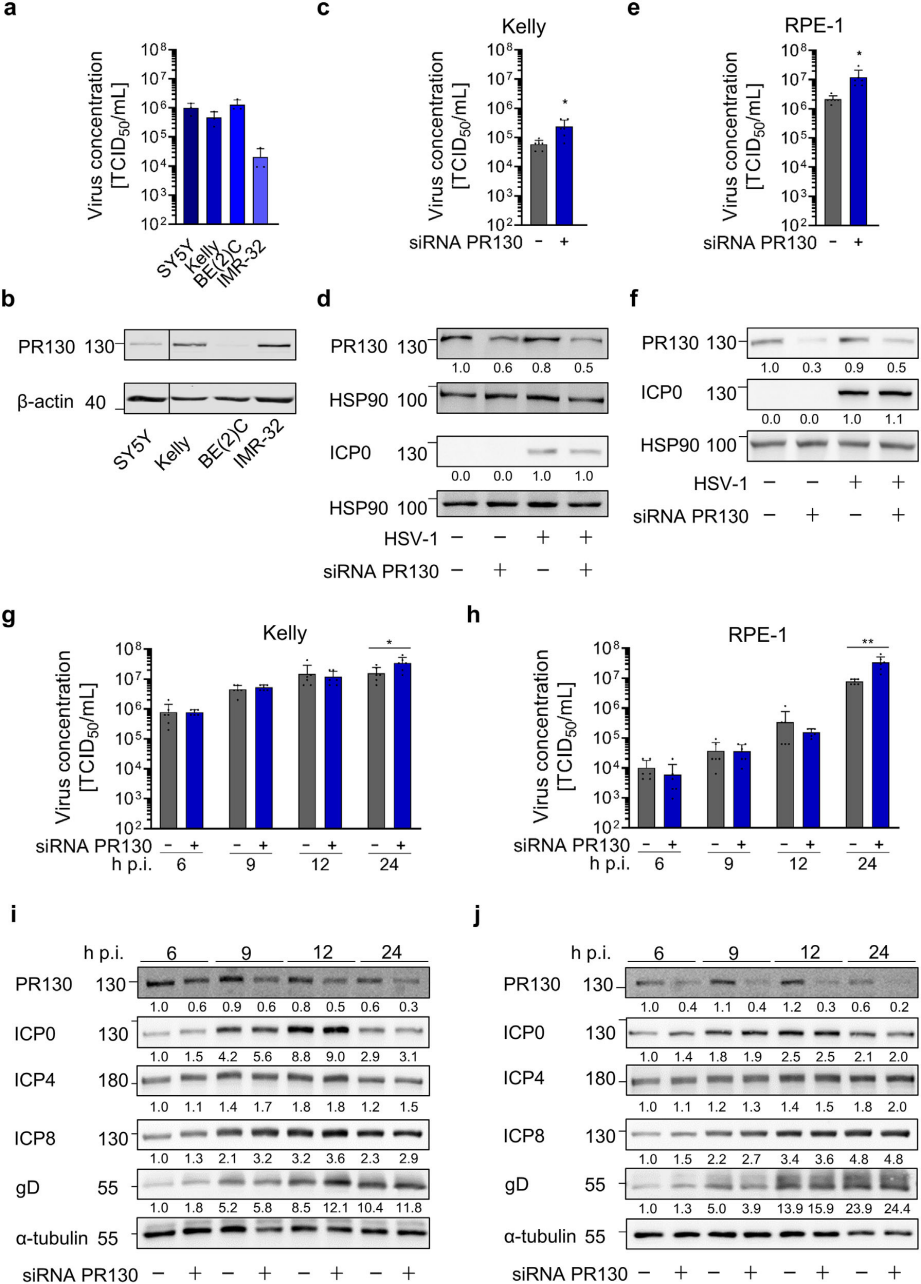
PR130 Antagonizes HSV‐1 Replication in Nerve and Retinal Epithelial Cells. (a) NB cells of 4 different origins (SY5Y, Kelly, BE(2)C, IMR32) were infected with HSV‐1/GFP using a MOI of 1. (b) The levels of PR130 and β‐actin were analyzed by immunoblot in NB cells. Numbers next to protein names indicate the molecular weight of the protein marker in kDa. (c,d) Kelly and (e,f) RPE‐1 cells were transfected with 100 pmol siRNA directed against PR130 (siRNA #1) or control siRNA for 48 h. Thereafter, cells were infected with HSV‐1/GFP using a MOI of (c,d) 1 or (e,f) 0.1. After medium change, cells were further incubated in media containing siRNA until 24 h p.i. (c,e) Virus concentrations were determined in the supernatants after 24 h p.i. Results of virus titrations are means (+SD) of TCID_50_ mL^−1^ values of 3 independent experiments, including 2 biological replicates. Statistical significance was analyzed by unpaired two‐tailed *t*‐tests against the control siRNA cells (*, *p* ≤ 0.05). (d,f) Immunoblots were used to analyze the presence of PR130, HSV‐1 ICP0, and the loading control HSP90 at 24 h p.i. Numbers next to protein names indicate the molecular weight of the protein marker in kDa. Numbers below represent the mean of 3 densitometric analyses relative to the loading control and normalized to the non‐infected or infected control cells (Figure S7d,e). (g,i) Kelly and (h,j) RPE‐1 cells were transfected with 100 pmol siRNA directed against PR130 (siRNA #1) or control siRNA for 48 h. Thereafter, cells were infected with HSV‐1/GFP using a MOI of (g,i) 1 or (h,j) 0.1. After medium change, cells were further incubated in media containing siRNA up to 24 h p.i. (g,i) Virus concentrations were determined in the supernatants at the indicated timepoints. Results of virus titrations are means (+SD) of TCID_50_ mL^−1^ values of 3 independent experiments, including 2 biological replicates. Statistical significance was analyzed by unpaired two‐tailed *t*‐tests against the control siRNA cells (*, *p* ≤ 0.05; **, *p* ≤ 0.01). (i,j) Immunoblots were used to analyze the presence of PR130, HSV‐1 ICP0, HSV‐1 ICP4, HSV‐1 ICP8, HSV‐1 gD, and the loading control α‐tubulin at the indicated timepoints. Numbers next to protein names indicate the molecular weight of the protein marker in kDa. Numbers below represent the mean of 3 densitometric analyses relative to the loading control and normalized to the normalized to the 6 h p.i. control cells (Figure S7f,g).

Neuroblastoma (NB) cell lines are frequently used as models for neuronal HSV‐1 infection [53, 54]. When we infected these cells with HSV‐1 and performed TCID_50_ tests, we noted that the cells’ abilities to produce HSV‐1 differed up to 60‐fold (Figure 3a). To test whether PR130 expression levels were linked to HSV‐1 replication, we conducted immunoblot analyses. We detected different levels of PR130 in the NB cell lines SY5Y, Kelly, BE(2)C, and IMR‐32 (Figure 3b). In NB cells with lower PR130 expression, we measured increased viral titers at 24 h p.i. (Figure 3a).

Comparing replication rates across different cell lines can be confounded by multiple variables. Accordingly, we studied whether reducing PR130 by RNAi in Kelly NB cells promoted HSV‐1 replication. We noted that a knockdown of PR130 with independent siRNAs increased the production of HSV‐1 (Figure 3c; Figure S3d). We could verify the downregulation of PR130 by the siRNAs in such cells. As in HCT116 and MIA PaCa‐2 cells, the PR130 status did not determine the accumulation of ICP0 in Kelly cells (Figure 3d).

To extend these analyses, we infected human retinal pigment epithelial RPE‐1 cells as model cells for ocular infections with HSV‐1. RPE‑1 cells require a lower viral concentration to achieve comparable levels of infection without a negative impact on vitality. As in Kelly cells, siRNAs against PR130 promoted HSV‐1 replication in RPE‐1 cells (Figure 3e; Figure S3e). We verified the downregulation of PR130 and an equal accumulation of ICP0 (Figure 3f). To exclude that different viral titers were caused by different cytotoxic effects of HSV‐1 on cells expressing different PR130 amounts and that the chosen MOIs were compatible with cell survival, we performed immunoblots assessing PARP1 integrity. HSV‐1 did not evoke apoptosis or necrosis associated processing of PARP1 in Kelly and RPE‐1 cells (Figure S3f,g).

To analyze how PR130 modulates HSV‐1 production and viral protein expression in such cells, we determined the concentrations of infectious HSV‐1 particles and performed immunoblots at 6–24 h of the viral replication cycle. There were significantly higher virus titers in Kelly and RPE‐1 cells that received siRNAs against PR130 at 24 h p.i., but no significant differences were seen at earlier timepoints (Figure 3g,h). Kelly and RPE‐1 cells accumulated viral proteins at different timepoints more efficiently when PR130 was decreased by RNAi (Figure 3i,j). This notion supports our hypothesis that an earlier release of HSV‐1 virions by HCT116^ΔPR130^ cells blunts the accumulation of higher levels of HSV‐1 proteins in cells (Figure 2b,c). Like in HCT116 cells, HSV‐1 attenuated PR130 in Kelly and RPE‐1 cells at 24 h p.i (Figure 3i,j).

These data show that PR130 attenuates HSV‐1 replication in cell types that resemble natural host cells of this virus.

### 2.4 Unbiased Proteomic Analyses Show How PR130 and HSV‐1 Regulate Protein Expression and Phosphorylation

**FIGURE 4:**
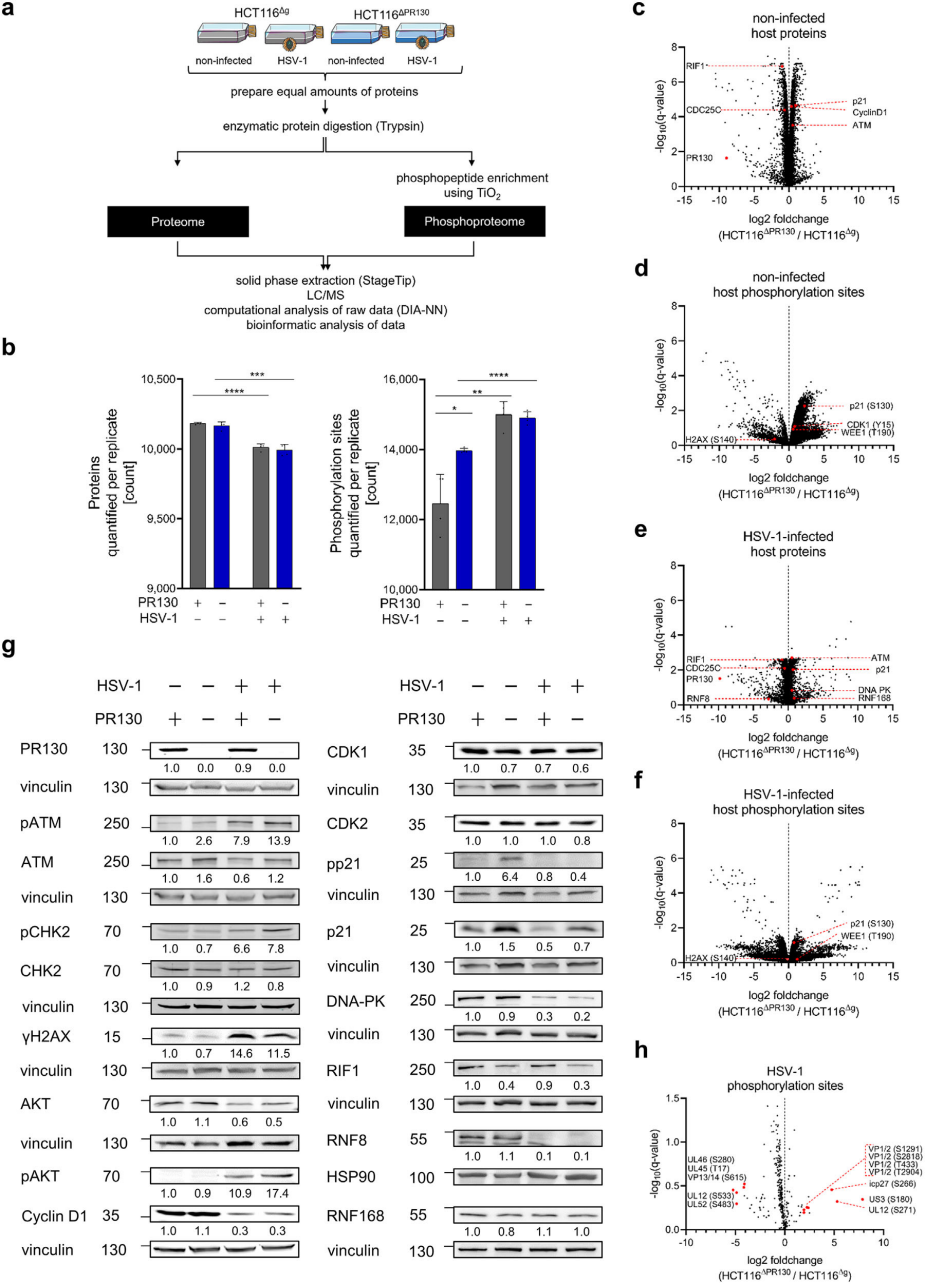
Mass Spectrometry‐based Proteomics and Phosphoproteomics Reveal Differentially Expressed Proteins and Phosphorylation Sites in PR130‐deficient HCT116 Cells. HCT116^Δg^ and HCT116^ΔPR130^ were infected with HSV‐1/KOS using a MOI of 2. At 6 h p.i., cells were lysed in guanidinium chloride buffer and subjected to mass spectrometry. (a) Schematic presentation of the analysis strategy. (b) Counts of quantified proteins and phosphorylation sites in HCT116^Δg^ and HCT116^ΔPR130^ cells. Statistical significance was analyzed by unpaired two‐tailed *t*‐tests against the control cells (*, *p* ≤ 0.05; **, *p* ≤ 0.01; ***, *p* ≤ 0.001; ****, *p* ≤ 0.0001) (c–f) Filtered host proteins. Values on the left: increase in HCT116^Δg^ cells and values on the right: increase in HCT116^ΔPR130^ cells. (g) Immunoblots were used to confirm the detection of differences in protein expression by proteomics 6 h p.i. Numbers next to protein names indicate the molecular weight of the protein marker in kDa. Numbers below represent the mean values of 3 densitometric analyses relative to the loading control and normalized to the non‐infected control cells (Figure S7h). (h) Mass spectrometry‐based proteomics and phosphoproteomics reveal differentially expressed HSV‐1 proteins and phosphorylation sites in HCT116 cells. Values on the left: increase in HCT116^Δg^ cells and values on the right: increase in HCT116^ΔPR130^ cells. (b–f,h) Data were collected with four replicates for each cell type.

Using unbiased proteomics, we searched for PR130‐regulated cellular factors that determine HSV‐1 propagation in HCT116^Δg^ and HCT116^ΔPR130^ cells. Uninfected and HSV‐1‐infected HCT116^Δg^ and HCT116^ΔPR130^ cells were subjected to total proteome and phosphoproteome analyses (Figure 4a). We observed similar protein levels (non‐infected 10 174 versus 10 129 and HSV‐1‐infected 9 979 vs 9 954) and phosphorylation patterns (non‐infected 12 459 vs 13 964 and HSV‐1‐infected 14 991 vs 14 897) in uninfected and HSV‐1‐infected HCT116^Δg^ cells and HCT116^ΔPR130^ cells (Figure 4b; Figure S4a–d). This assay further showed that significantly less proteins were detected in infected HCT116^Δg^ cells and HCT116^ΔPR130^ cells, which corresponds to a transcriptional repression of host genes by HSV‐1 [55]. Irrespective thereof, total host protein phosphorylation levels were significantly increased upon HSV‐1 infection. Significantly higher phosphorylation levels in resting HCT116^ΔPR130^ cells than in HCT116^Δg^ cells corresponds to PR130 being part of the PP2A complex and that HCT116^ΔPR130^ cells did not adapt with decreased phosphorylation to the loss of PR130 (Figure 4b). Divergently expressed proteins and phosphorylation sites in HCT116^ΔG^ and HCT116^ΔPR130^ cells include several cell cycle regulators, checkpoint kinases, and NHEJ proteins in the uninfected (Figure 4c,d) and HSV‐1‐infected states (Figure 4e,f).

Based on these findings, we carried out immunoblotting. Uninfected and HSV‐1‐infected HCT116^ΔPR130^ cells had higher phosphorylated ATM (p‐ATM, S1981) and total ATM levels than HCT116^Δg^ cells (Figure 4g). The phosphorylation of the ATM target protein CHK2 at T68 (pCHK2) was increased in infected HCT116^ΔPR130^ cells compared to HCT116^Δg^ cells, verifying stronger activation of the ATM‐CHK2 axis in such cells. This was not linked to a general induction of cellular DNA replication stress, as evidenced by a comparable accumulation of its biochemical marker ɣH2AX (Figure 4g). ATM phosphorylates AKT at S473 and HSV‐1 induces this phosphorylation [51, 56, 57]. Consistent with the augmented activation of ATM in HCT116^ΔPR130^ cells, we found a strong accumulation of phosphorylated AKT (pAKT, S473) in HSV‐1‐infected HCT116^ΔPR130^ cells. This was associated with decreased AKT levels (Figure 4g). Proteomics showed a downregulation of Cyclin D1 upon infection with HSV‐1 (Figure S4a,c), which we could confirm by immunoblot (Figure 4g). The cyclin‐dependent kinases CDK1 and CDK2 were reduced to a lesser, respectively no extent by HSV‐1 (Figure 4g). Our proteome analyses and immunoblots illustrate that HCT116^ΔPR130^ cells have higher expression levels of p21 in the uninfected and the HSV‐1‐infected states and enhanced phosphorylation of p21 (S130) than HCT116^Δg^ cells in the uninfected state (Figure 4c–g).

DNA‑PK contributes to NHEJ and restricts the replication of HSV‐1 [23, 25]. Consistent with DNA‐PK being a target of ICP0 [24, 45], DNA‐PK levels were attenuated in both cell types upon HSV‐1 infection (Figure 4g). Replication timing regulatory factor 1 (RIF1) promotes NHEJ and disfavors the homologous recombination (HR) DNA repair pathway [58]. There are lower levels of RIF1 in uninfected and HSV‐1 infected HCT116^ΔPR130^ cells than in HCT116^Δg^ cells (Figure 4g). This notion is coherent with a negative impact of NHEJ on HSV‐1 replication [23, 25] and the higher titers of HSV‐1 in HCT116^ΔPR130^ cells when compared with HCT116^Δg^ cells (Figure 1a). Further NHEJ proteins include RNF8 and RNF168 [59]. Proteomics detected that RNF8 was equally expressed in non‐infected HCT116^ΔPR130^ and HCT116^Δg^ cells and it was more reduced in HCT116^ΔPR130^ than in HCT116^Δg^ cells after HSV‐1 infection (Figure 4c,e; Figure S4a,c). RNF8 was undetectable after HSV‐1 infection by immunoblot in both cell types (Figure 4g). We also analyzed RNF168 as protein with similar levels in proteomics of HCT116^ΔPR130^ and HCT116^Δg^ cells. Immunoblots confirmed the equal expression of RNF168 in both cell types (Figure 4g).

Proteomics additionally showed a PR130‐dependent control of HSV‐1 proteins and their phosphorylation. We detected 69 HSV‐1 proteins and similar phosphorylation events of HSV‐1 proteins (339 versus 331) in HCT116^Δg^ and HCT116^ΔPR130^ cells (Figure 4h; Figure S4e). These comprise phosphorylation sites of the immediate early protein ICP27, the serine/threonine kinase US3, and the exonuclease UL12 in HCT116^ΔPR130^ cells. We also observed that four different phosphorylation sites of the largest tegument protein of HSV‐1, VP1/VP2 were enriched in such cells (Figure 4h). Other phosphorylation events ‐ in the HSV‐1 tegument proteins VP13/14 (UL47) and UL46, the exonuclease UL12, the DNA Primase UL52, and the envelope protein UL45 were decreased in HCT116^ΔPR130^ cells (Figure 4h). This may be explained by an increased liberation of viral progeny by such cells than by HCT116^Δg^ cells (Figure 1a).

These results disclose that PR130 controls expression and phosphorylation of selected host and HSV‐1 proteins.

### 2.5 CDK2 is Required for HSV‐1 Replication

**FIGURE 5:**
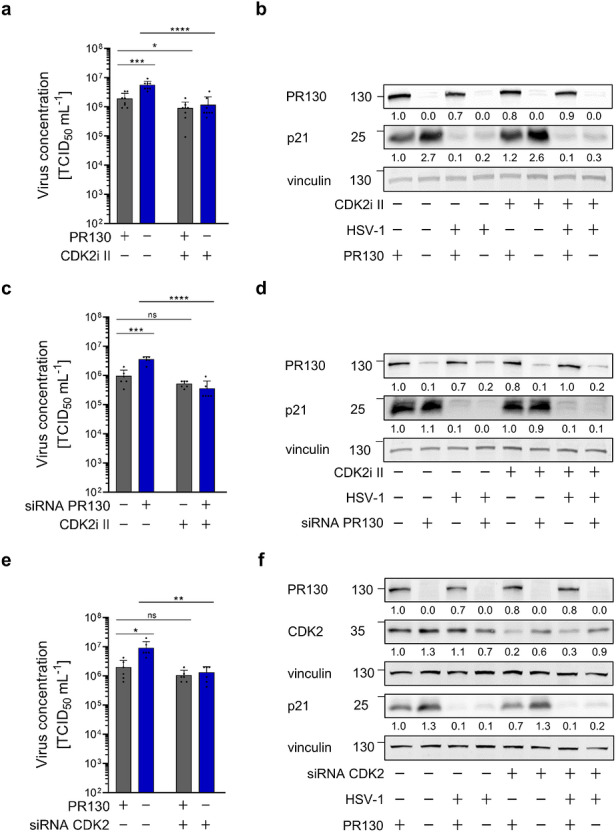
CDK2 Inhibition Suppresses HSV‐1 Replication. (a, b) HCT116^Δg^ and HCT116^ΔPR130^ cells were treated with 5 µM CDK2i or DMSO as solvent control for 24 h and then infected with HSV‐1/GFP using a MOI of 1. After medium change, cells were further incubated in CDK2i‐containing medium. (a) Results of virus titrations are means (+SD) of TCID_50_ mL^−1^ values of 3 independent experiments, including 2 biological replicates. (b) Immunoblot was done as indicated. (c,d) RPE‐1 cells were transfected with 100 pmol siRNA (siRNA #1), treated with 5 µM CDK2i or DMSO, and infected with HSV‐1/GFP using a MOI of 1. After medium change, cells were further incubated in siRNA‐ or CDK2i‐containing medium. (c) Results of virus titrations are means (+SD) of TCID_50_ mL^−1^ values of 3 independent experiments, including 2 biological replicates. (d) Immunoblot was done as indicated. (e, f) HCT116^Δg^ and HCT116^ΔPR130^ cells were transfected with 60 pmol siRNA and infected with HSV‐1/GFP using a MOI of 1. After a medium exchange, cells were further incubated in siRNA‐containing medium. (e) Results of virus titrations are means (+SD) of TCID_50_ mL^−1^ values of 3 independent experiments, including 2 biological replicates. (f) Immunoblot was done as indicated. (a,c,e) Virus concentrations were determined in supernatants 24 h p.i. Statistical significance was analyzed by unpaired two‐tailed *t*‐tests against the control cells (ns, not significant; *, *p* ≤ 0.05; ***, *p* ≤ 0.001; ****, *p* ≤ 0.0001). (b,d,f) Immunoblots were used to analyze the levels of PR130, p21, CDK2, and the loading control vinculin 24 h p.i. Numbers next to protein names indicate the molecular weight of the protein marker in kDa. Numbers below represent the mean of 3 densitometric analyses relative to the loading control and normalized to untreated non‐infected control cells (Figure S7i–k).

We reported that HCT116 cells with and without PR130 underwent different types of cell cycle arrest upon drug‐induced DNA replication stress [49]. Because infection of HCT116^Δg^ and HCT116^ΔPR130^ cells with HSV‐1 induces similar cell cycle alterations (Figure 2a), cell cycle variations cannot explain our findings for anti‐HSV‐1 functions of PR130. The replication of HSV‐1 correlates with the activity of CDK2 [20, 60] which catalyzes the phosphorylation of p21 at S130. Thus, the increased phosphorylation of p21 at S130 in HCT116^ΔPR130^ cells indicates increased CDK2 activation, which might in turn explain why such cells produce more HSV‐1 than HCT116 cells expressing PR130. To assess this hypothesis, we treated HCT116^Δg^ and HCT116^ΔPR130^ cells with a CDK2 inhibitor (CDK2i). Subsequently, cell cultures received HSV‐1 and virus concentrations were determined in supernatants after 24 h p.i. CDK2i suppressed HSV‐1 replication in both HCT116^Δg^ and HCT116^ΔPR130^ cells, but with more significant effects in HCT116^ΔPR130^ cells. Inhibition of CDK2 ceased the higher production of HSV‐1 by HCT116^ΔPR130^ cells to a level equal to HSV‐1 production in HCT116^Δg^ cells (Figure 5a).

Immunoblotting showed that CDK2i did not alter PR130 levels and the HSV‐1‐induced depletion of p21 significantly (Figure 5b). CDK2i also did not disrupt cell cycle profiles (Figure S5). These data exclude that CDK2i prevents HSV‐1 replication through cell cycle disruption.

To substantiate these results, we depleted PR130 in RPE‐1 cells and applied CDK2i and HSV‐1. We noted that CDK2i suppressed HSV‐1 replication in RPE‐1 cells with reduced PR130 to a level equal to HSV‐1 production in RPE‐1 cells that received control siRNA molecules (Figure 5c). Immunoblotting showed that the CDK2i did not alter PR130 expression and did not halt the HSV‐1‐induced depletion of p21 (Figure 5d).

We could corroborate these results using RNAi‐based targeting CDK2. An siRNA‐mediated knockdown of CDK2 demonstrated that CDK2 activity was required for HSV‐1 replication (Figure 5e). Immunoblot confirmed the reduction of CDK2 and additionally demonstrated that CDK2 was more stable in HCT116^ΔPR130^ cells than in HCT116^Δg^ cells. As with the CDK2i, siRNA against CDK2 did not alter p21 and its HSV‐1‐induced reduction (Figure 5f).

These data disclose that the higher replication of HSV‐1 in cells lacking PR130 is linked to a druggable activity of CDK2.

### 2.6 PR130 Modulates p21 and ATM as Cell‐intrinsic Regulators of HSV‐1 Replication

**FIGURE 6:**
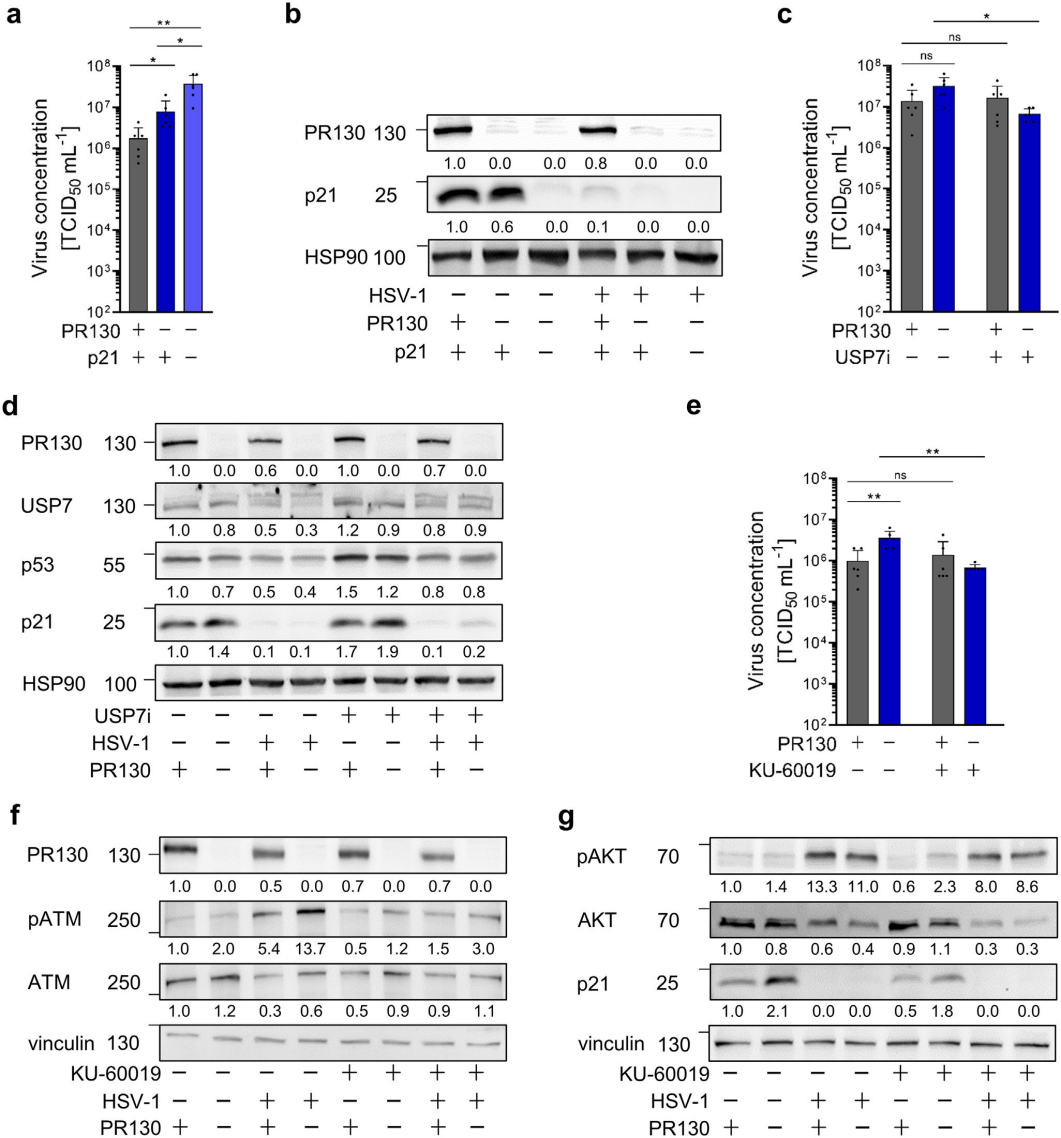
PR130 and Its Target Proteins p21 and ATM Control HSV‐1 Replication. (a,b) HCT116^∆g^, HCT116^ΔPR130^, and HCT116^ΔPR130Δp21^ cells were infected with HSV‐1/GFP using a MOI of 1. (c–g) HCT116^Δg^ and HCT116^ΔPR130^ cells were treated with (c,d) 2 µM USP7 inhibitor (USP7i) or DMSO as a solvent control or (e–g) 1 µM KU‐60019 or DMSO as a solvent control for 24 h. Thereafter, cells were infected with HSV‐1/GFP using a MOI of 1. After medium change, cells were further incubated in inhibitor‐containing medium. (a,c,e) Virus concentrations were determined in the supernatants at 24 h p.i. Results of virus titrations are means (+SD) of TCID_50_ mL^−1^ values of 3 independent experiments, including 2 biological replicates. Statistical significance was analyzed by unpaired two‐tailed *t*‐tests against the control cells (ns, not significant; *, *p* ≤ 0.05; **, *p* ≤ 0.01; ****, *p* ≤ 0.0001). (b,d,f,g) Immunoblots were used to analyze the levels of PR130, p21, USP7, p53, pATM (S1981), ATM, pAKT (S473), AKT, and the loading controls HSP90, or vinculin 24 h p.i. Numbers next to protein names indicate the molecular weight of the protein marker in kDa. Numbers below represent the mean of 3 densitometric analyses relative to the loading control and normalized to the untreated non‐infected control cells (Figure S7l–o).

Dependent on its concentration and its posttranslational modifications, p21 inhibits or aids in the formation of CDK‐cyclin complexes [61, 62]. We noted higher levels of p21 and pS130‐p21 in HCT116^ΔPR130^ cells than in HCT116^Δg^ cells, but a depletion of p21 in HSV‐1‐infected cells (Figures 4c–g and 5b,d,f). To assess the relevance of p21 stringently, we generated HCT116^ΔPR130^ cells with an additional elimination of p21 by CRISPR‐Cas9. We infected such HCT116^ΔPR130Δp21^ cells with HSV‐1 and measured infectious HSV‐1 particles in supernatants at 24 h p.i. We noted a further increase of viral titers in HCT116^ΔPR130Δp21^ cells compared to HCT116^ΔPR130^ cells (Figure 6a). We verified the combined PR130/p21‐deficiency of HCT116^ΔPR130Δp21^ cells and the reduction of p21 and PR130 by HSV‐1 in cognate HCT116^Δg^ cells by immunoblot (Figure 6b).

We evaluated these results with an approach that targets the ubiquitin‐specific protease‐7 (USP7, also termed herpesvirus‐associated ubiquitin‐specific protease, HAUSP) which modulates the induction of p21 by the transcription factor p53 [63, 64, 65]. We also chose USP7 for additional analyses as it is a target of HSV‐1 [66]. Proteome analyses illustrate that after HSV‐1 infection both HCT116^Δg^ and HCT116^ΔPR130^ cells have reduced levels of USP7 (Figure S4a,c). To assess the relevance of USP7 during HSV‐1 infection, we treated HCT116^Δg^ and HCT116^ΔPR130^ cells with non‐toxic concentrations of an USP7 inhibitor (USP7i, USP7‐797) (Figure S6a) and infected them with HSV‐1. USP7i reduced HSV‐1 titers in HCT116^ΔPR130^ cells but not in HCT116^Δg^ control cells (Figure 6c). Immunoblots verified that HSV‐1 infection reduced USP7 (Figure 6d). Coherent with the reported activation and stabilization of p53 and p21 upon USP7 inhibition [63, 64, 65] our immunoblots illustrated higher levels of p53 and p21 in HCT116^Δg^ and HCT116^ΔPR130^ cells after treatment with USP7i (Figure 6d). Hence, a drug‐induced accumulation of p21 in this setting is linked to HSV‐1 suppression in HCT116^ΔPR130^ cells.

Our previous studies [49] and our new results (Figure 4) show that in addition to p21, PR130 and HSV‐1 infection modulate the expression and phosphorylation‐dependent activation of checkpoint kinases. To assess the relevance of ATM signaling during HSV‐1 infection, we treated HCT116^Δg^ and HCT116^ΔPR130^ cells with non‐toxic concentrations of the ATM inhibitor KU‐60019 (Figure S6b) and infected such cells with HSV‐1. KU‐60019 reduced the production of infectious HSV‐1 particles in HCT116^ΔPR130^ cells but not in HCT116^Δg^ cells (Figure 6e). Immunoblots verified that KU60019 diminished activation of ATM at S1981 and pAKT in HSV‐1‐infected cells (Figure 6f). We tested if this effect of KU‐60019 might be explained by altered p21 levels. KU60019 did not alter p21 expression in resting and HSV‐1‐infected HCT116^Δg^ and HCT116^ΔPR130^ cells (Figure 6g), indicating independent relevance of ATM and p21 for HSV‐1 replication.

The outcome of these experiments discloses that PR130 controls the replication of HSV‐1 through its target proteins p21 and ATM.

## 3 Discussion

This work suggests that PR130 is a newly identified host protein regulating HSV‐1 and a newly identified target protein of HSV‐1. Thus, the PP2A‐PR130 complex adds to the few findings which PP2A B‐type subunits determine viral propagation and cell fate [67, 68]. These include the interaction of human T cell leukemia virus 1 and 2, Marburg‐, Ebola‐ and Cuevaviruses with the cellular PP2A‐B56 complex [69, 70]. In addition, the PP2A B‐type subunit B55α binds to the adenovirus E4orf4 protein to induce apoptotic cell death and cell cycle arrest [71, 72]. Regarding the family of the *Herpesviridae*, the Kaposi sarcoma‐associated herpesvirus protein LANA interacts with the B56ε subunit of PP2A. This disrupts the holoenzyme and consequently alters the phosphorylation of nuclear proteins [73]. Furthermore, PP2A is activated in cells upon infection with HSV‐1. This attenuates the pro‐survival serine/threonine kinases mitogen‐activated protein kinase and extracellular signal‐regulated kinase, and thereby the phosphorylation‐dependent inactivation of the pro‐apoptotic BCL2 protein BAD in hepatoma cells [74]. HSV‐1 also modulates PP2A to control the IFN regulatory factor‐3 [75]. We disclose that upon HSV‐1 infection PR130 modulates the expression and phosphorylation levels of proteins that cells require for cell cycle control, DNA damage sensing, and DNA repair to restrict HSV‐1 replication.

Another key finding of this work is that PR130 modulates HSV‐1 replication through its impact on cells before and upon infection. The mechanism underlying the control of HSV‐1 by PR130 in resting cells relies on the activity of CDK2 and its crosstalk with p21. The involvement of CDK2, which is most active in S phase [62], reflects that DNA viruses replicate mainly in S phase. The fact that HCT116^ΔPR130^ cells have more cells in S phase than HCT116^Δg^ cells can accordingly contribute to the increased viral production by HCT116^ΔPR130^ cells. Nonetheless, alterations in cell cycle progression cannot explain the need of HSV‐1 replication for CDK2. In various cells a decrease of the CDK2 substrate p21, which controls DNA replication speed and promotes cell cycle arrest in G1 phase and G2/M phase [76], is linked to the replication of HSV‐1. Consistent herewith, delayed S phase progression by inhibiting the p21 interaction partner proliferating cell nuclear antigen inhibits HSV‐1 DNA synthesis [77]. In addition to p21, the CDK inhibitory kinase WEE1 [76] may affect HSV‐1 replication. Our phosphoproteome data show that both pWEE1 (T190) and pp21 (S130) are regulated differently in cells with and without PR130. A depletion of WEE1 by HSV‐1 was noted [78]. We demonstrate that CDK2 inhibition impairs viral replication. CDK2 inhibitors are used in clinical trials against viral diseases and cancer [79, 80]. Further studies are underway to define if the HSV‐1‐induced depletion of WEE1 and p21 arrests cells in the S phase, in which the highest concentrations of nucleotides are available for viral replication.

The apoptotic caspase substrate and DNA repair protein PARP1 was neither cleaved in PR130‐deficient cells nor in PR130 siRNA‐treated cells that were infected with HSV‐1 at the MOIs that we chose. These data verify that reduced metabolic activities of HSV‐1‐infected HCT116 cells are not linked to apoptosis. As reported [57, 81, 82, 83, 84, 85], HSV‐1 induced the phosphorylation of AKT which facilitates virus entry, replication, and reactivation. This occurred to higher levels in PR130‐deficient cells. AKT phosphorylates downstream targets, among others p21, which lessens the affinity of p21 to CDK2. Consequently, the negative effect of p21 on CDK2 is decreased [86]. AKT inhibitors were found to be effectively antiviral in mouse and in vitro HSV‐1 infection models [87, 88]. These datasets are consistent with our finding that the p21‐CDK2 axis controls the replication of HSV‐1. We unravel that this axis is a downstream target of the PP2A‐PR130 complex and that inhibition of USP7 induces p53‐p21 signaling and suppress HSV‐1. Targeting USP7 represents a promising antiviral strategy, demonstrating efficacy against HSV‐1, Epstein‐Barr virus, the human immunodeficiency virus HIV1, adenoviruses, and coronaviruses [89, 90, 91, 92, 93].

The appearance of viral genomes in the nucleus induces cellular DNA damage responses [94]. ATM and ATR kinases are activated after HSV‐1 infection and are required for its efficient replication [27]. We recently described PR130 as upstream regulator of ATM, CHK1, WEE1, and p21, and therefore as a cell cycle modulator after DNA replication stress [49]. This work discloses that HSV‐1 induces activating ATM phosphorylation and that PR130 counteracts this posttranslational modification. HSV‐1 benefits from such ATM signaling. HSV‐1 replicates significantly better in HCT116^ΔPR130^ cells than in HCT116^Δg^ cells and significantly less in HCT116^ΔPR130^ cells that are treated with an ATM inhibitor. ATM affects DNA damage repair pathway choice between HR and NHEJ, which restricts HSV1 replication [35, 36, 95]. Thus, the positive effect of ATM on HSV‐1 replication might stem from mechanisms favoring HR. ATM and DNA‐PK were reported to be antagonistic regulators of HSV‐1 [24, 45]. DNA‐PK and RIF1 suppress HR and enhance NHEJ activity [58, 96, 97]. The reduced expression of RIF1 in HCT116^ΔPR130^ cells, and the decrease of DNA‐PK following HSV1 infection support this hypothesis. Both processes are associated with more efficient HSV1 replication.

Regarding HSV‐1 proteins, we found that several phosphorylation sites were upregulated in HCT116^ΔPR130^ cells. These proteins are linked to the pathogenicity of HSV‐1. The serine/threonine kinase US3 phosphorylates viral and cellular targets and increases HSV‐1 virulence by promoting replication, translation, assembly, and egress, and acts as anti‐apoptotic protein [98, 99, 100, 101]. The activity of the UL12 exonuclease is essential for producing viral DNA and thus for generating infectious viral particles [102, 103]. The largest tegument protein of HSV‐1, VP1/VP2, is conserved across members of the *Herpesviridae*. It is essential for HSV‐1 replication by regulating several steps in the life cycle: entry, capsid transport, virion assembly, formation of mature virions, and microtubule‐mediated transport of capsids [104, 105, 106]. Thus, the PP2A‐PR130 complex is a regulator of both host and HSV‐1 proteins.

### 3.1 Limitations

This work discloses that PR130 affects the HSV‐1 replication cycle through distinct host proteins in various cell systems. We have not yet analyzed how PR130 affects viral proteins, their phosphorylation, and how this may affect HSV‐1 production. The detection of the underlying, often very transient interactions of PR130‐PP2A with such target proteins requires sophisticated assays. This also applies for the molecular mechanisms how HSV‐1 decreases PR130 upon prolonged infection. Additional research is required to show if and how PR130 affects other DNA and RNA viruses.

## 4 Conclusions

HSV‐1 is common and endemic throughout the world. This work is the first report on how the PP2A subunit PR130 regulates the replication and spread of HSV‐1 and on how such pathways can be addressed with experimental and clinically used small molecules. Therefore, our research is relevant from virological and pharmacological perspectives. It will be interesting to see how effective these agents are against the global burden of HSV‐1.

## 5 Methods

### 5.1 Chemicals

CDK2 inhibitor II (CAS no. 222035‐13‐4) was obtained from Santa Cruz Biotechnology (Heidelberg, Germany), KU‐60019 (S1570) from Selleckchem (Cologne, Germany) and USP7‐797 (HY‐136910) from Hycultec GmbH (Beutelsbach, Germany). Compounds were diluted in DMSO and further in culture media to keep final DMSO concentrations below 0.1%.

### 5.2 Cell Lines and Viruses

We described the generation and characterization of CRISPR‐Cas9‐based PR130‐depleted HCT116 cells (RRID:CVCL_0291) in [49]. Human retinal pigment epithelial cells (hTERT RPE‐1) (RRID:CVCL_4388) (CRL‐4000, ATCC, Manassas, Virginia, USA; provided by Prof. T. Hofmann, Mainz, Germany) and MIA PaCa‐2 cells (RRID:CVCL_0428) (CRL‐1420, ATCC; provided by Prof. G. Schneider, Göttingen, Germany) were cultured in DMEM (AC‐LM‐0012, Anprotec, Bruckberg, Germany) supplemented with 5% fetal bovine serum (FBS, P30‐3031, PANBiotech, Aidenbach, Germany). The neuroblastoma cell lines Kelly (RRID:CVCL_2092) (ACC355, DSMZ, Braunschweig, Germany), SY5Y (RRID:CVCL_0019) (CRL‐2266, ATCC), BE(2)C (RRID:CVCL_0529) (CRL‐2268, ATCC), and IMR‐32 (RRID:CVCL_0346) (CCL‐127, ATCC) were provided by Prof. I. Oehme, Heidelberg, Germany. These were cultivated in DMEM supplemented with 10% FBS and 1% nonessential amino acids (AC‐AS‐0011, Anprotec, Bruckberg, Germany). We have summarized the isolation and cultivation of murine pancreatic ductal adenocarcinoma (PDAC) cultures in [52]. The HSV‐1 strains used in this work are the recombinant HSV‐1*17K with a clinically relevant glutamine‐70‐lysine (E70K) substitution in the viral deoxyribonucleic acid polymerase and an insertion of GFP [11, 12] (in the following labeled as HSV‐1/GFP) as well as the laboratory strain HSV‐1/KOS. The clinical isolates of HSV‐1, 1076/1998, 1123/1999, and 598/2002 were collected from human patients [107]. All viruses were propagated in green monkey kidney cells, Vero76 (RRID:CVCL_0603) (CRL‐1587, ATCC), which were cultivated in DMEM supplemented with 10% FBS. HSV‐1 infections occur independent of the sexes. Accordingly, we use human female cells (RPE‐1, Kelly, and SY5Y) as well as male cells (HCT116, IMR‐32, BE(2)C, and MIA PaCa‐2). These were tested free of mycoplasma by enzymatic testing (MycoStrip, rep‐mys‐50, InvivoGen, Toulouse, France).

### 5.3 CRISPR‐Cas9

HCT116^ΔPR130^ cells were transfected with a total of 2 400 ng of 2 sgRNAs against the p21‐encoding *CDKN1A* gene locus (IDT, Hs.Cas9.CDKN1A.1.AA, Hs.Cas9.CDKN1A.1.AC, Leuven, Belgium) (Sequences: *GTCGAAGTTCCATCGCTCACGGG*; *TTCTGACGGACATCCCCAGCCGG*) and 6 250 ng recombinant Cas9‐GFP protein (CAS9GFPPRO, Merck, Darmstadt, Germany) according to the CRISPRMAX Reagent Cas9 nuclease transfection protocol (ThermoFisher Scientific, CMAX00001) for 24 h. MIA PaCa‐2 cells were transfected with a total of 2 400 ng of 2 sgRNAs against the PR130‐encoding *PPP2R3A* gene locus (sequences were chosen according to [49]) and the recombinant Cas9‐GFP protein transfection mixture. Control cells received the Cas9 protein without sgRNAs. GFP‐positive cells were obtained as single cell clones by flow cytometry. Efficient knockdown was confirmed by immunoblotting.

### 5.4 Viral Infection and TCID_50_ Titration

Per well, 2 × 10^6^ HCT116, 3 × 10^5^ RPE‐1, or 1 × 10^6^ Kelly cells were seeded in 6‐well plates 24 h prior to infection. Cells were washed once with PBS and infected with the indicated multiplicity of infection (MOI) of HSV‐1 in DMEM without FCS for 1 h at 37°C and 5% CO_2_. The MOI was chosen dependent on how efficient HSV‐1 replicates in the various cell systems, with the aim for its strong replication with minimal cell death. After aspiration of supernatants, cells were incubated for up to 23 h in DMEM with 5% FCS (or 10% FCS for Kelly cells). Thereafter, supernatants were collected to determine viral titers. Cells were collected for immunoblotting or flow cytometry.

Generally, 1.5 × 10^4^ Vero76 per well cells were seeded in 96‐well plates 24 h prior to titration. Serial 1:10 dilutions of viral‐containing supernatants in DMEM without FCS were added to the cells. After 3 d at 37°C and 5% CO_2_, cytopathic effects were visualized microscopically and TCID_50_ values were calculated [108].

### 5.5 siRNA and Plasmid Transfection

Knockdown of PR130 in Kelly and RPE‐1 cells was performed by transfecting 100 pmol siRNA for PR130 (#1; s10975, ThermoFisher Scientific, Dreieich, Germany, and #2; HY‐RS11015, Hycultec GmbH, Beutelsbach, Germany) or corresponding amounts of non‐targeting control siRNA (sc‐44231, Santa Cruz Biotechnology, Heidelberg, Germany) with 2.5 µL Lipofectamine2000 (11668019, ThermoFisher Scientific, Dreieich, Germany) according to the manufacturer's protocol. After 24 h, this transfection procedure was repeated. After a total period of 48 h, Kelly and RPE‐1 cells were infected. Knockdown of CDK2 in HCT116 cells was performed by transfecting 60 pmol siCDK2 (HY‐RS02374, Hycultec GmbH, Beutelsbach, Germany) or corresponding amounts of non‐targeting control siRNA with 2.5 µL Lipofectamine2000 according to the manufacturer's protocol. After a transfection period of 24 h, HCT116 cells were infected. After 1 h, cells were incubated in the transfection mixture until 24 h post‐infection (p.i.). An effective knockdown was confirmed by immunoblotting.

For complementing HCT116^ΔPR130^ cells with PR130, we used two different plasmids encoding PR130. These are pcDNA3‐HA‐PR130 [49] and pcDNA‐PR130‐GFP which was a kind gift from Dr. D. Gül, Mainz University Medical Center, Germany. The latter contains the sequence for green fluorescent protein (GFP) from *Aequorea Victoria* in frame with the sequence coding PR130 (amplified out of pcDNA3‐HA‐PR130 and inserted using BamHI‐NheI cutting sites). Both plasmids were verified by whole plasmid sequencing (Eurofins, Cologne, Germany). HCT116 cells were transfected with 3 µg plasmid DNA and 7 µL Lipofectamine2000 (11668019, ThermoFisher Scientific, Dreieich, Germany) according to the manufacturer's protocol. After a transfection period of 24 h, HCT116 cells were infected. After 1 h, cells were incubated in the transfection mixture until 24 h p.i. An effective transfection was confirmed by immunoblotting.

### 5.6 Flow Cytometry Analysis

After the indicated infection time, cells were detached using trypsin, collected with supernatants, and washed with PBS after centrifugation (5 min 3000 rpm 4°C). Cells were resuspended in 100 µL cold PBS and fixed for at least 1 h with 2 mL cold 80% ethanol at −20°C. After centrifugation, cells were resuspended in 200 µL PBS, mixed with 10 µL PI (556463, BD Biosciences, Heidelberg, Germany), and analyzed with flow cytometry (FACSLyric, BD Biosciences, Heidelberg, Germany). Cell cycle distributions were examined using ModFit LT 6.0 software (RRID: SCR_016106) (Verity Software House, Maine, USA).

### 5.7 Immunoblot and Antibodies

For immunoblotting, cells were detached with trypsin, washed, and pellets were resuspended in Triton lysis buffer (20 mM Tris‐HCl, pH 7.4; 137 mM NaCl; 10% glycerol; 1% Triton X‐100; 2 mM EDTA; 50 mM sodium glycerophosphate; 20 mM sodium pyrophosphate; 5 µg mL^−1^ aprotinin; 0.2 mM pefablock; 5 µg mL^−1^ leupeptin; 1 mM sodium vanadate; and 5 mM benzamidine). Lysates were incubated at 4°C for 30 min. After centrifugation (15 min 20 000 x g, 4°C), total protein content of all lysates was determined (Protein Assay Dye Reagent, BioRad, Feldkirchen, Germany) and equalized. Samples were supplemented with 5x Laemmli buffer (10% SDS, 50% glycerol, 25% β‐mercaptoethanol, 0.02% bromophenol blue, 312 mM Tris, pH 6.8) and heated for 10 min at 95°C. Equal volumes were loaded on SDS‐PAGE and blotted on 0.2 µm nitrocellulose membranes. Membranes were developed in a Fusion FX6.Edge system (Vilber Lourmat, Eberhardzell, Germany) and analyzed with the Fusion software Evolution‐Capt (RRID:SCR_023580) (Vilber Lourmat, Eberhardzell, Germany) using either ECL Western blotting substrate (Pierce, ThermoFisher Scientific, Dreieich, Germany) or fluorescent secondary antibodies. For the quantification of the signals, Fiji Is Just ImageJ (RRID:SCR_002285) (FIJI V. 2.16.0/1.54p) was applied [109].

The following antibodies were used in this study: anti‐p21 (ab109520; RRID:AB_10860537), anti‐p‐S1981‐ATM (ab81292; RRID:AB_1640207), anti‐ATM (ab32420; RRID:AB_725574), anti‐AKT (ab32505; RRID:AB_722681), anti‐CyclinD1 (ab134175; RRID:AB_2750906), anti‐DNA‐PKcs (ab32566; RRID:AB_731981; against the catalytic subunit of DNA‐PK) from Abcam (Cambridge, United Kingdom); anti‐PARP1 (611039; RRID:AB_398352) from BD Biosciences (Heidelberg, Germany); anti‐HSP90 (4877; RRID:AB_2233307), anti‐p‐T68‐CHK2 (2661; RRID:AB_331479), anti‐CHK2 (2662; RRID:AB_2080793), anti‐α‐tubulin (2125; RRID:AB_2619646) from Cell Signaling Technology (WZ Leiden, The Netherlands); anti‐p‐S139‐H2A.X (05‐636; RRID:AB_309864) from Merck (Darmstadt, Germany); anti‐PR130 (NBP1‐87233; RRID:AB_11030500) from Novus Biologicals (Bio‐Techne GmbH, Wiesbaden Nordenstadt, Germany); anti‐HSV‐1‐ICP0 (sc‐53070; RRID:AB_673704), anti‐Vinculin (sc‐73614; RRID:AB_1131294), anti‐β‐actin (sc‐47778; RRID:AB_626632), anti‐CDK1 (sc‐8395; RRID:AB_627225), anti‐CDK2 (sc‐6248; RRID:AB_627238), anti‐ERK2 (sc‐1647; RRID:AB_627547), anti‐HAUSP (sc‐137008; RRID:AB_2214163), anti‐p53 (sc‐126; RRID:AB_628082), anti‐RNF8 (sc‐271462; RRID:AB_10648902), anti‐RNF168 (sc‐101125; RRID:AB_2180105) from Santa Cruz Biotechnology (Heidelberg, Germany); anti‐p‐S130‐p21 (PA5‐12644; RRID:AB_10979980), anti‐p‐S473‐AKT (44‐621G; RRID:AB_2533699), anti‐RIF1 (A300‐569A; RRID:AB_669804) from ThermoFisher Scientific (Dreieich, Germany). Secondary antibodies HRP goat anti‐mouse (926‐80010; RRID:AB_2721263), HRP goat anti‐rabbit (926‐80011; RRID:AB_2721264), IRDye 680RD goat anti‐mouse (926‐68070; RRID:AB_10956588), IRDye 680RD goat anti‐rabbit (926‐68071; RRID:AB_10956166), and IRDye 800CW goat anti‐rabbit (926‐32211; RRID:AB_621843) were obtained from Li‐COR Biotechnology (Bad Homburg, Germany).

### 5.8 Wide‐Field Fluorescence Microscopy

We recently described this method in detail [110]. Briefly, per well, 0.2 × 10^6^ HCT116 cells were seeded in 24‐well plates supplemented with sterile glass cover slips (CarlRoth, Karlsruhe, Germany, #1; 0.13 – 0.16 mm thickness) 24 h prior to infection and were left either uninfected or were infected with a MOI of 1 of HSV‐1/GFP in DMEM without FCS for 1 h at 37°C and 5% CO_2_. After 24 h, cells were fixed with 3.7% formaldehyde (Sigma‐Aldrich, Taufkirchen, Germany)/PBS for 15 min, permeabilized with 0.1% Triton‐X100 (Sigma‐Aldrich, Taufkirchen, Germany) /PBS for 15 min, and blocked with 3% BSA (CarlRoth, Karlsruhe,Germany)/PBS for 30 min. Hoechst 33342 (Sigma‐Aldrich, Germany) was diluted in 3% BSA/PBS and incubated for 1 h at room temperature to visualize cellular DNA. Mounting medium (Ibidi, Gräfelfing, Germany) was added to cover cell layers. All slides were stored at 4°C. The z‐stacks were acquired as 14‐bit images using an AXIO Observer.Z1 microscope (Zeiss, Jena, Germany) equipped with ApoTome.2 (Zeiss, Germany), Axiocam 503 mono (Zeiss, Jena, Germany), an HXP 120 V light source (Leistungselektronik Jena GmbH, Germany), and Zen blue version 2.6 (RRID:SCR_013672) (Zeiss, Jena, Germany). For image acquisition, the following filter sets (Zeiss, Jena, Germany) were used for the respective dyes: 38HE (ex: 450–490 nm, em: 500–550 nm) for AF488, and 49 (ex: 335–383 nm, em: 420–470 nm) for DAPI. Images were acquired using a Plan‐Apochromat 20x/0.8 NA air objective (Zeiss, Jena, Germany) and a fixed voxel size of 0.227 µm in xy and 0.490 µm in z, with light intensities and exposure times as indicated in Table 1. After acquisition, images were directly deconvolved in Zen blue with phase error correction and a deconvolution strength of 6. For image display, deconvolved z‐stacks were maximum intensity projected in Zen blue and the dynamic ranges were adjusted as described in Table 1.

**TABLE 1:** Image acquisition parameters and displayed dynamic ranges of Figure 1d.

Component	chromophore	Light intensity [%]	Exposure time [ms]	Displayed Dynamic Range
HSV‐1	GFP	100	1 000	100 – 1 000
Nucleus	Hoechst33342	65	100	100 – 600

The GFP fluorescence signals were quantified based on maximum intensity projections using the inbuilt measure function of FIJI (RRID:SCR_002285) (V. 2.16.0/1.54p) [109]. The mean fluorescence intensity of five pictures were plotted and means (+SD) were calculated.

### 5.9 DNA Isolation and Real Time PCR

DNA isolation was performed using the QIAamp DNA Mini kit (ID. 51304, Qiagen, Hilden, Germany) according to the manufacturer`s protocol. QuantiNova SYBR green PCR kit (ID. 208056, Qiagen, Hilden, Germany) was used for the real time PCR. In total, 8 µL master mix (5 µL 2xSYBR green, 0.75 µL 10 µM forward primer, 0.75 µL 10 µM reverse primer, 1.5 µL RNase‐free water) and 50 ng DNA were multiplied in a 72‐well RotorGene (Qiagen, Hilden, Germany) in the following cycling conditions: 2 min 95°C, 40 cycles of 5 s 95°C and 10 s 60°C. The melting curves were obtained by a stepwise temperature increase (1°C every 5 s) from 60°C to 95°C. The Cq‐values were normalized to GAPDH.

The following primer pairs were used: GAPDH (CTCTGCTCCTCCTGTTCGAC; CAATACGACCAAATCCGTTGAC), RS1_ICP4 (GCCAGAGACAGACCGTCAGA; TGGGAAAAAGGACAGGGACG), UL54_ICP27 (CGCCAAGAAAATTTCATCGAG; ACATCTTGCACCACGCCAG), UL29_ICP8 (CACCAGGTTGCGCATCAG; CTGCATACGGTGGTGAACAAC), UL41_VHS (GGACATCCGCGACGAAAAC; AGAAACCTGTCGGCGATATCAG), US6_gD (ACGGTTTACTACGCCGTGTT; TGTAGGGTTGTTTCCGGACG), UL48_VP16 (CCGGGTCCGGGATTTACC; CTCGAAGTCGGCCATATCCA).

### 5.10 Viral Infection for Proteomics

In each 10 cm cell culture dish, 10^7^ HCT116 cells were seeded 24 h prior to the experiment. Cells were left either uninfected or were infected with a MOI of 2 of HSV‐1 in DMEM w/o FCS for 1 h at 37°C and 5% CO_2_. After aspiration of supernatants, cells were incubated in DMEM with 5% FCS until 6 h p.i. Thereafter, cells were detached from plates using trypsin, washed twice with PBS, and lysed in 1 mL guanidinium chloride buffer (6 M guanidinium chloride, 50 mM Tris HCl, pH 8.0). Samples were incubated at 90°C with shaking for 15 min, followed by sonication for 10 min in an ice‐cold bath. After centrifugation (15 min 20 000 x g, 4°C), total protein contents were determined (Protein Assay Dye Reagent, BioRad, Feldkirchen, Germany) and diluted to 5 µg µL^−1^ with lysis buffer. Samples were stored at ‐80°C until further processing.

### 5.11 Enzymatic Protein Digestion

Samples were processed using the SP3 approach [111]. Briefly, 200 µg of proteins per sample were reduced with 5 mM dithiothreitol (DTT), alkylated in 15 mM iodoacetamide in the dark and quenched with 5 mM DTT. Enzymatic protein digestion was performed using trypsin (protein:protease ratio = 200:3) at 37°C overnight. Following acidification by formic acid, the peptides were purified by solid phase extraction in a C18 cartridge.

### 5.12 Phosphopeptide Enrichment

Phosphopeptide enrichment was performed in a microcolumn set‐up using TiO_2_ [112]. Desalted peptides were eluted directly in loading buffer (80% acetonitrile, 2% trifluoroacetic acid (TFA), 1 M lactic acid [113, 114]) and added to a microcolumn packed with 5 mg of TiO_2_ beads (Titansphere 5‐µm; GL Sciences, Tokyo, Japan), followed by centrifugation at 100 × g. The microcolumn was then washed once with loading buffer and twice with wash buffer (80% acetonitrile, 0.1% TFA). Elution of the enriched peptides was performed twice by centrifugation at 500 × g in elution buffer (50% acetonitrile, 1% ammonia). Thereafter, acetonitrile and ammonia were removed using a centrifugal evaporator at 45°C. The resulting peptide solution was acidified with TFA and purified using a solid phase extraction in a StageTip format [115].

### 5.13 Liquid Chromatography Tandem Mass Spectrometry

Peptides were separated via an in‐house packed 45 cm analytical column (inner diameter: 75 µm; ReproSil‐Pur 120 C18‐AQ 1.9 µm silica particles, Dr. Maisch GmbH, Ammerbuch, Germany) on a Vanquish Neo UHPLC system (Thermo Fisher Scientific, Dreieich, Germany). Online reverse‐phase chromatography was conducted using a 40 min non‐linear gradient of 1.6%–32% acetonitrile with 0.1% formic acid at a nanoflow rate of 300 nL min^−1^. The eluted peptides were sprayed directly by electrospray ionization into an Orbitrap Astral mass spectrometer (Thermo Fisher Scientific, Dreieich, Germany). Mass spectrometry (MS) was conducted in data‐independent acquisition (DIA) mode. Full MS spectra were acquired in the Orbitrap analyzer in profile mode (scan range: 350 to 1 050 m/z; resolution: 120 000, target value: 3 × 10^6^, maximum injection time: 20 ms). The precursor ions were isolated for fragmentation at a window width of 4 Th over the range of 350 to 1 050 m/z. The fragment ions were recorded in centroid mode in the Astral analyzer via higher energy collision dissociation (HCD; normalized collision energy: 26%, scan range: 150 to 2 000 m/z, target value: 5 × 10^4^, maximum injection time: 5 ms, default charge state: +2).

### 5.14 Mass Spectrometry Data Analysis

Raw data files were processed in DIA‐NN software (version 1.9.2) (RRID:SCR_022865) [116] in library‐free mode. An in silico predicted spectral library was generated in DIA‐NN from the UniProt reference proteomes of *H. sapiens* (release 2024_03; 104 626 entries), HSV‐1 strain 17 (taxonomy ID: 10299; release 2024_05; 77 entries), and a list of common contaminants.

For the proteome data, the MS/MS spectra were searched against the predicted library using the following settings: Trypsin/P protease specificity, one missed cleavage allowed, maximum 1 variable modification, carbamidomethylation of cysteine as fixed modification, methionine oxidation as variable modification, peptide length range 7–30, precursor charge range 2–5, MBR and heuristic protein inference switched on, “Genes” for protein inference, “RT‐dependent” for cross‐run normalization and “IDs, RT and IM Profiling” for library generation. The following settings were used to filter the identified precursors: Lib.Q.Value ≤ 0.01, Lib.PG.Q.Value ≤ 0.01. Thereafter, the MaxLFQ algorithm [117] was applied to the “Precursor.Normalised” quantity using the DIA‐NN R package for protein quantification. The LFQ intensities were further normalization by median‐centering at the protein group level based on the detected human proteins.

For the phosphoproteome data, the MS/MS spectra were searched against the predicted library using the following settings: Trypsin/P protease specificity, one missed cleavage allowed, maximum 3 variable modifications, carbamidomethylation of cysteine as fixed modification, methionine oxidation and phosphorylation at STY residues as variable modifications, peptide length range 7–30, precursor charge range 2–5, MBR and heuristic protein inference switched on, scoring in “peptidoforms” activated, “Genes” for protein inference, cross‐run normalization switched off and “IDs, RT and IM Profiling” for library generation. The direct output file of DIA‐NN “phosphosites_90.tsv”, corresponding to phosphosites with a localization probability greater than 0.9, was used for downstream analysis.

Differential expression analysis was performed using the limma package in R [118]. For each comparison, proteins or phosphosites were filtered to retain only those detected in at least 3 out of the 4 replicates in either group of each comparison. Following imputation of the missing intensity values, a linear model was fitted to assess the difference between the two groups for each protein or phosphosite, with adjustment for multiple testing using the Benjamini‐Hochberg approach [119]. The log_2_ fold change and the significance of the difference were displayed on a volcano plot. Only proteins or phosphosites with a minimum log_2_ fold change of 1 and an FDR‐adjusted *p* value (q value) lower than 0.05 were considered differentially regulated.

The mass spectrometry proteomics data have been deposited to the ProteomeXchange Consortium via the PRIDE partner repository with the dataset identifier PXD067327 [120].

### 5.15 WST‐1 Assay

HCT116 cells (100 µL/well) were seeded at a density of 3 × 10^5^ mL^−1^ in 96‐well plates 24 h prior to infection. Cells were washed once with PBS and remained non‐infected or were infected with a MOI of 1 of HSV‐1 for 24 h. At 22 h p.i., 10 µL of WST‐1 (5015944001, Merck, Darmstadt, Germany) were added to each well and cultures were further incubated for 2 h at 37°C. Absorbance values were measured at 450 nm using a FLUOstar Omega plate reader (BMG Labtech, Ortenberg, Germany).

### 5.16 MTT Assay

We recently described this assay [121]. Briefly, HCT116 cells (100 µL/well) were seeded at a density of 3 × 10^5^ mL^−1^ in 96‐well plates 24 h prior to treatment. Cells were washed with PBS and incubated for 24 h with freshly prepared substance dilutions in DMEM. Finally, 25 µL of 5 mg mL^−1^ MTT (Sigma‐Aldrich, Taufkirchen, Germany) were added for another 2 h. Thereafter, supernatants were removed, DMSO was added to lyse cells, and metabolic activity was determined via absorbance at OD 562 nm using a FLUOstar Omega plate reader (BMG Labtech, Ortenberg, Germany).

### 5.17 Statistics

Statistical analyses were performed with GraphPad Prism 8 for Windows (RRID:SCR_002798) (GraphPad Software, Boston, Massachusetts, USA), which are described in the figure legends, respectively. Unpaired two‐tailed *t*‐test was used for comparisons between two groups, and 2way ANOVA with Dunnett's multiple comparisons test was used for the MTT assays. All data are shown as the mean with standard deviation (SD). n, represents the number of independent experiments. In all analyses, 95% confidence intervals (95% CI) were calculated, and *p*‐values less than 0.05 were considered statistically significant. All statistically *p*‐value is represented by a symbol (* *p* < 0.05, ** *p* < 0.01, *** *p* < 0.001, **** *p* ≤ 0.0001). All *p*‐values are listed in Table S1.

### 5.18 Data Availability

Submission details: Project Name: Proteome and phosphoproteome profiling of HCT116 colon cancer cells with PP2A subunit PR130 deletion and herpes simplex virus HSV‐1 infection, Project accession: PXD067327.

## Author Contributions

J.J.: data curation, formal analysis, investigation, methodology, software, validation, visualization, writing – original draft, writing – review & editing. C.F.J.: formal analysis, investigation, methodology, writing – review & editing. A.N.: formal analysis, investigation, methodology. M.B.: formal analysis, investigation, methodology. A.O.M.: formal analysis, investigation, writing – review & editing. M.D.: formal analysis, software, writing – review & editing. J.‐X.C.: formal analysis, investigation, methodology, resources, software, writing – review & editing. W.B.: resources, writing – review & editing. C.E.: resources, validation, writing – review & editing. A.H.: conceptualization, data curation, funding acquisition, project administration, supervision, validation, writing – original draft, writing – review & editing. O.H.K.: conceptualization, data curation, funding acquisition, project administration, resources, supervision, validation, writing – original draft, writing – review & editing.

## Funding

This work was made possible with funding from the German Research Foundation/Deutsche Forschungsgemeinschaft (DFG) grant HE2910/16‐1 and KR2291/17‐1, DFG‐project number 502534123 to A.H. and O.H.K. Work done in the group of O.H.K. is additionally funded by the DFG grant KR2291/12‐2, DFG‐project number 445785155; KR2291/14‐1, DFG‐project number 469954457; KR2291/15‐1, DFG‐project number 495271833; KR2291/16‐1, DFG‐project number 496927074; DFG‐project number 393547839 – SFB 1361, sub‐project 11, 316213987‐SFB 1278, sub‐project D02; the Walter Schulz Stiftung; the Brigitte und Dr. Konstanze Wegener‐Stiftung (project 110); the Deutsche José Carreras Leukämie‐Stiftung (project DJCLS 09 R/204); the H.W. & J. Hector Stiftung project M 2419; and the Dr. Werner Jackstädt‐Stiftung. Work done in the group of C.E. is additionally funded by the DFG‐project number 316213987 – SFB 1278, sub‐project D02.

## Conflicts of Interest

O.H.K. declares the patents “The use of molecular markers for the preclinical and clinical profiling of inhibitors of enzymes having histone deacetylase activity, WO/2004/027418”, “Novel HDAC6 inhibitors and their uses, WO2016020369A1”, and “Synthesis, pharmacology and use of new and selective FMS‐like tyrosine kinase 3 (FLT3) inhibitors, WO2019/034538”, and advisory consultant activity for BASF, Ludwigshafen, Germany. The substances that are covered in these patents are not those that are shown in the submitted manuscript. Thus, there are no direct conflicts of interest. All other authors declare that they have no conflicts of interest.

## Supporting information




**Supporting File 1**: advs75687‐sup‐0001‐FigureS1‐S7.docx.

**Supporting File 2**: advs75687‐sup‐0002‐TableS1.docx.

**Supporting File 3**: advs75687‐sup‐0003‐Data.zip.

**Supporting File 4**: advs75687‐sup‐0004‐FigureLegends.pdf.

## Data Availability

The data that support the findings of this study are available from the corresponding author upon reasonable request.
