# Supplementary material for: The Host Cell Factor Phosphatase‐2A Subunit PR130 Restricts Replication of Herpes Simplex Virus Type‐1
Source: Adv Sci (Weinh). 2026 May 15;13(44):e23697. doi: 10.1002/advs.202523697 (PMC13335924; doi:10.1002/advs.202523697)
Supplement: Supplementary file 2 [file ADVS-13-e23697-s001.docx]

Table S1. P values for each figure.

| Figure | Sample | Comparison | p value |
| --- | --- | --- | --- |
| 1a | HCT116 | Δg vs. ΔPR130 | 0.0474 |
| 1c | Clinical isolate 1 | Δg vs. ΔPR130 | 0.0036 |
|  | Clinical isolate 2 | Δg vs. ΔPR130 | 0.0109 |
| 1e | HCT116 | Δg vs. ΔPR130 | 0.0224 |
| 1f | HCT116 | ctrlΔg vs. ctrlΔPR130 | 0.0243 |
|  |  | ctrlΔg vs. HA-PR130Δg | 0.0163 |
|  |  | ctrlΔg vs. GFP-PR130Δg | 0.0012 |
|  |  | ctrlΔPR130 vs. HA-PR130ΔPR130 | 0.0017 |
|  |  | ctrlΔPR130 vs. GFP-PR130ΔPR130 | 0.0005 |
| 2a | 1 h p.i G2/M | Non-infected Δg vs. non-infected ΔPR130 | <0.0001 |
|  | 1 h p.i S | Non-infected Δg vs. non-infected ΔPR130 | 0.0009 |
|  | 1 h p.i G0/G1 | Non-infected Δg vs. non-infected ΔPR130 | <0.0001 |
|  | 1 h p.i G2/M | infected Δg vs. infected ΔPR130 | <0.0001 |
|  | 1 h p.i S | infected Δg vs. infected ΔPR130 | 0.0074 |
|  | 1 h p.i G0/G1 | infected Δg vs. infected ΔPR130 | <0.0001 |
|  | 1 h p.i G2/M | Non-infected Δg vs. infected Δg | 0.0389 |
|  | 1 h p.i G0/G1 | Non-infected Δg vs. infected Δg | <0.0001 |
|  | 1 h p.i G2/M | Non-infected ΔPR130 vs. infected ΔPR130 | 0.0022 |
|  | 1 h p.i S | Non-infected ΔPR130 vs. infected ΔPR130 | 0.0134 |
|  | 1 h p.i G0/G1 | Non-infected ΔPR130 vs. infected ΔPR130 | 0.0016 |
|  | 6 h p.i G2/M | Non-infected Δg vs. non-infected ΔPR130 | <0.0001 |
|  | 6 h p.i G0/G1 | Non-infected Δg vs. non-infected ΔPR130 | <0.0001 |
|  | 6 h p.i G2/M | infected Δg vs. infected ΔPR130 | <0.0001 |
|  | 6 h p.i G0/G1 | infected Δg vs. infected ΔPR130 | 0.0008 |
|  | 6 h p.i G2/M | Non-infected Δg vs. infected Δg | <0.0001 |
|  | 6 h p.i G0/G1 | Non-infected Δg vs. infected Δg | <0.0001 |
|  | 6 h p.i G2/M | Non-infected ΔPR130 vs. infected ΔPR130 | 0.0031 |
|  | 6 h p.i S | Non-infected ΔPR130 vs. infected ΔPR130 | 0.0025 |
|  | 6 h p.i G0/G1 | Non-infected ΔPR130 vs. infected ΔPR130 | 0.0018 |
|  | 12 h p.i G2/M | Non-infected Δg vs. non-infected ΔPR130 | <0.0001 |
|  | 12 h p.i G0/G1 | Non-infected Δg vs. non-infected ΔPR130 | <0.0001 |
|  | 12 h p.i G2/M | infected Δg vs. infected ΔPR130 | 0.0125 |
|  | 12 h p.i G2/M | Non-infected Δg vs. infected Δg | <0.0001 |
|  | 12 h p.i S | Non-infected Δg vs. infected Δg | 0.0001 |
|  | 12 h p.i G0/G1 | Non-infected Δg vs. infected Δg | 0.0061 |
|  | 12 h p.i G2/M | Non-infected ΔPR130 vs. infected ΔPR130 | <0.0001 |
|  | 24 h p.i G2/M | Non-infected Δg vs. non-infected ΔPR130 | <0.0001 |
|  | 24 h p.i G0/G1 | Non-infected Δg vs. non-infected ΔPR130 | <0.0001 |
|  | 24 h p.i G2/M | Non-infected Δg vs. infected Δg | <0.0001 |
|  | 24 h p.i S | Non-infected Δg vs. infected Δg | 0.0002 |
|  | 24 h p.i G0/G1 | Non-infected Δg vs. infected Δg | 0.0462 |
|  | 24 h p.i G2/M | Non-infected ΔPR130 vs. infected ΔPR130 | 0.0003 |
|  | 24 h p.i S | Non-infected ΔPR130 vs. infected ΔPR130 | 0.0486 |
| 2b | 9 h p.i. | Δg vs. ΔPR130 | 0.0237 |
|  | 24 h p.i. | Δg vs. ΔPR130 | 0.0233 |
| 2d | *UL29* 6 h p.i. | Δg vs. ΔPR130 | 0.0015 |
|  | *UL29* 9 h p.i. | Δg vs. ΔPR130 | 0.0027 |
|  | *UL29* 12 h p.i. | Δg vs. ΔPR130 | 0.0376 |
|  | *UL41* 6 h p.i. | Δg vs. ΔPR130 | 0.0017 |
|  | *UL48* 6 h p.i. | Δg vs. ΔPR130 | <0.0001 |
|  | *UL54* 6 h p.i. | Δg vs. ΔPR130 | <0.0001 |
|  | *RS1* 6 h p.i. | Δg vs. ΔPR130 | <0.0001 |
|  | *US6* 6 h p.i. | Δg vs. ΔPR130 | 0.0018 |
| 3c | Kelly | siRNA ctrl vs. siRNA PR130 | 0.0178 |
| 3d | RPE-1 | siRNA ctrl vs. siRNA PR130 | 0.0198 |
| 3g | Kelly 24 h p.i | siRNA ctrl vs. siRNA PR130 | 0.0414 |
| 3i | RPE-1 24 h p.i. | siRNA ctrl vs. siRNA PR130 | 0.0050 |
| 4b | HCT116 proteins | Non-infected Δg vs. infected Δg | <0.0001 |
|  |  | Non-infected ΔPR130 vs. infected ΔPR130 | 0.0003 |
|  | HCT116 phosphorylations | Non-infected Δg vs. non-infected ΔPR130 | 0.0115 |
|  |  | Non-infected Δg vs. infected Δg | 0.0015 |
|  |  | Non-infected ΔPR130 vs. infected ΔPR130 | <0.0001 |
| 5a | HCT116 | Untreated Δg vs. untreated ΔPR130 | 0.0003 |
|  |  | Untreated Δg vs. treated Δg | 0.0278 |
|  |  | Untreated ΔPR130 vs. treated ΔPR130 | <0.0001 |
| 5c | RPE-1 | Untreated siRNA ctrl vs. untreated siRNA PR130 | 0.0001 |
|  |  | Untreated siRNA PR130 vs. treated siRNA PR130 | <0.0001 |
| 5e | HCT116 | siRNA ctrl Δg vs. siRNA ctrl ΔPR130 | 0.0154 |
|  |  | siRNA ctrl ΔPR130 vs. siRNA CDK2 ΔPR130 | 0.0088 |
| 6a | HCT116 | Δg vs. ΔPR130 | 0.0464 |
|  |  | ΔPR130 vs. ΔPR130Δp21 | 0.0125 |
|  |  | Δg vs. ΔPR130 Δp21 | 0.0034 |
| 6c | HCT116 | Untreated ΔPR130 vs. treated ΔPR130 | 0.0110 |
| 6e | HCT116 | Untreated Δg vs. untreated ΔPR130 | 0.0056 |
|  |  | Untreated ΔPR130 vs. treated ΔPR130 | 0.0014 |
| S1d | 24 h p.i. | Δg vs. ΔPR130 | 0.0497 |
| S2b | MIA PaCa-2 | Δg vs. ΔPR130 | 0.0060 |
| S2d | PDAC | 3250 vs. S821 | 0.0018 |
|  |  | 3250 vs. 8296 | <0.0001 |
| S3d | Kelly | siRNA ctrl vs. siRNA PR130 | 0.0071 |
| S3e | RPE-1 | siRNA ctrl vs. siRNA PR130 | 0.0451 |
| S6a | HCT116 Δg | 0 vs. 10 µM | <0.0001 |
|  |  | 0 vs. 7.5 µM | 0.0182 |
|  | HCT116 ΔPR130 | 0 vs. 10 µM | 0.0019 |
| S6b | HCT116 Δg | 0 vs. 10 µM | <0.0001 |
|  |  | 0 vs. 7.5 µM | <0.0001 |
|  |  | 0 vs. 5 µM | 0.0004 |
|  |  | 0 vs. 2.5 µM | 0.0040 |
|  | HCT116 ΔPR130 | 0 vs. 10 µM | <0.0001 |
|  |  | 0 vs. 7.5 µM | <0.0001 |
|  |  | 0 vs. 5 µM | <0.0001 |
|  |  | 0 vs. 2.5 µM | 0.0036 |
