# Supplementary material for: The Host Cell Factor Phosphatase‐2A Subunit PR130 Restricts Replication of Herpes Simplex Virus Type‐1
Source: Adv Sci (Weinh). 2026 May 15:e23697. Online ahead of print. doi: 10.1002/advs.202523697 (PMC13335924; doi:10.1002/advs.202523697)
Supplement: Supplementary file 1 — Supporting File 1: advs75687‐sup‐0001‐FigureS1‐S7.docx. [file ADVS-9999-e23697-s001.docx]

**The Host Cell Factor Phosphatase-2A Subunit PR130 Restricts Replication of Herpes Simplex Virus Type-1**

Johannes Jungwirth^1,2^, Christoph F. Jacob^1^, Alexandra Nguyen^2^, Mandy Beyer^2^, Andreas O. Mieland^2^, Mario Dejung^3^, Jia-Xuan Chen^3^, Walburgis Brenner^4^, Christina Ehrhardt^1^, Andreas Henke^1,#,^*, Oliver H. Krämer^2,#,^*

^1^ Section of Experimental Virology, Institute of Medical Microbiology, Center for Molecular Biomedicine (CMB), Jena University Hospital, Jena, Germany

^2^ Institute of Toxicology, University Medical Center of the Johannes Gutenberg University Mainz, Mainz, Germany

^3^ Institute of Molecular Biology gGmbH (IMB), Proteomics Core Facility, Mainz, Germany

^4^ Department of Obstetrics and Gynecology, University Medical Center of the Johannes Gutenberg University Mainz, Mainz, Germany

^#^equal last authors

*Corresponding authors

Prof. Dr. Oliver H. Krämer

Building 905 / 15^th^ floor / Room 1536

Obere Zahlbacher St. 67

55131 Mainz

Germany

**Correspondence:** Oliver Krämer ([okraemer@uni-mainz.de](mailto:okraemer@uni-mainz.de), tumor biology) /

Andreas Henke ([andreas.henke76@outlook.de](mailto:andreas.henke76@outlook.de), virology)

### Figure legends to supplementary figures


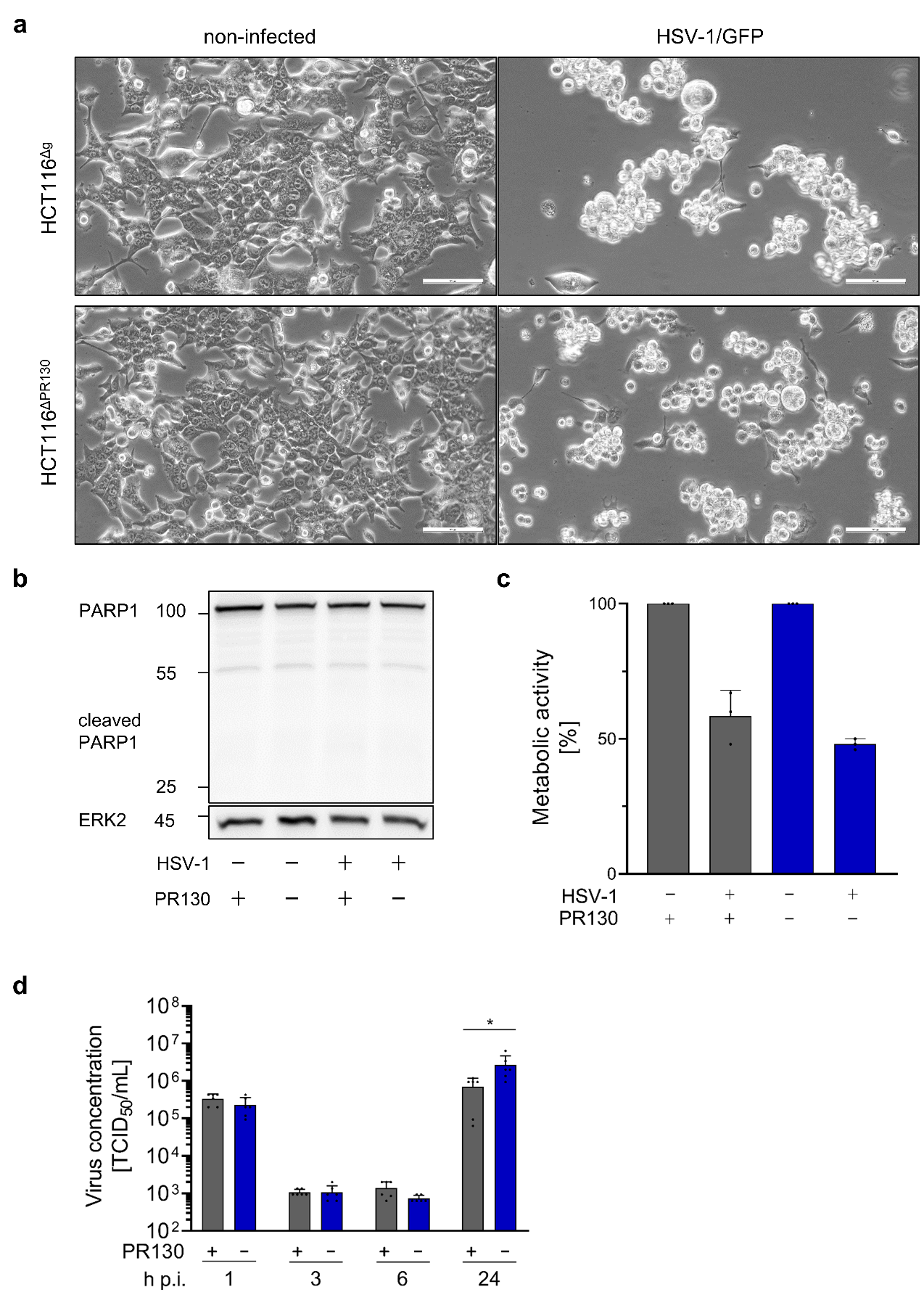


**Figure S1.** HCT116^Δg^ and HCT116^ΔPR130^ cells were infected with (a,b) HSV-1/GFP or (c) HSV-1/KOS using a MOI of 1. (a) HCT116^Δg^ and HCT^ΔPR130^ cells reveal similar morphology at 24 h p.i., as visualized by an AxioVert.A1 microscope (10-fold magnification) and monitored by an Axiocam 208 color camera. Representative images are shown. Scale bars represent 50 μm. (b) Immunoblots were used to analyze the levels of PARP1 and the loading control ERK2 at 24 h p.i. Numbers next to protein names indicate the molecular weight of the protein marker in kDa. (c) Metabolic activity of HSV-1/KOS-infected cells were analyzed by WST-1 tests at 24 h p.i. Means (+SD) of 10 independent parallels are demonstrated, with non-infected controls arbitrarily set to 100%. (d) HCT116^Δg^ and HCT116^ΔPR130^ cells were infected with HSV-1/GFP using a MOI of 1. Virus concentrations were determined in the supernatants at 24 h p.i. Results of virus titrations are means (+SD) of TCID_50_ mL^-1^ values of 3 independent experiments, including 2 biological replicates. Statistical significance was analyzed by unpaired two-tailed t-tests against the control cells (*, p ≤ 0.05).

**
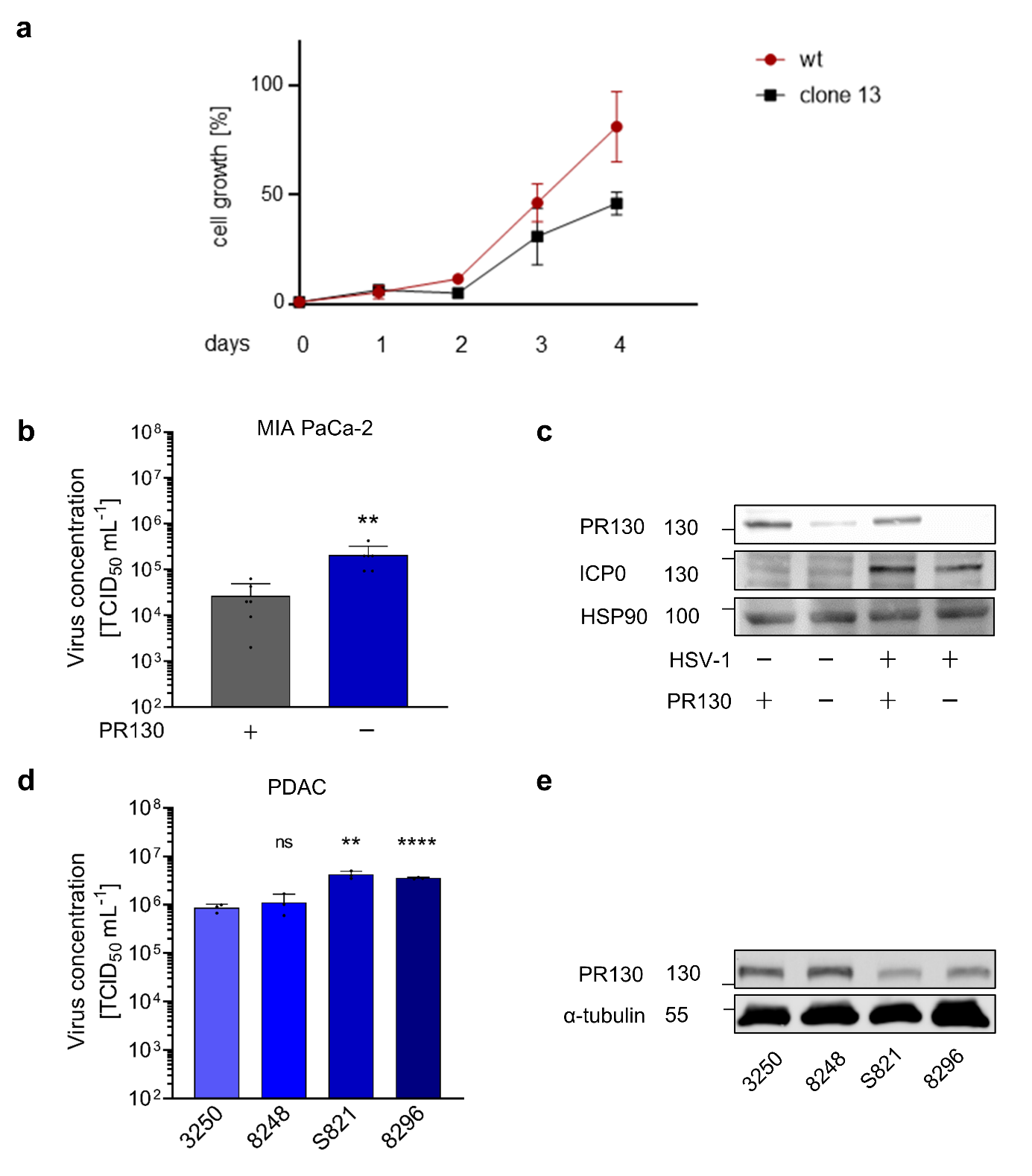
**

**Figure S2.** MIA PaCa-2^ΔPR130^ cells were analyzed. (a) The selected clone #13 initially proliferates like wild-type MIA PaCa-2 cells but show a delayed long-term growth. (b,c) MIA PaCa-2^Δg^, and MIA PaCa-2^ΔPR130^ cells were infected with HSV-1/KOS using a MOI of 1. (b) Total virus concentrations were determined by TCID_50_ titrations at 24 h p.i. Results of virus titrations are means (+SD) of TCID_50_ mL^-1^ values of 3 independent experiments, including 2 biological replicates. (c) Immunoblots were used to analyze levels of PR130, HSV-1 ICP0 and the loading control HSP90. (e,f) Murine PDAC short-term cell cultures show an increased HSV-1 replication dependent on PR130 expression. (d) PDAC cells were infected with HSV-1/KOS using a MOI of 1. Virus concentrations were determined in the supernatants at 24 h p.i. Results of virus titrations are means (+SD) of TCID_50_ mL^-1^ values of 3 independent experiments, including 2 biological replicates. (e) Immunoblots indicate differential expression of PR130 in 4 non-infected PDAC cultures. (b,d) Statistical significance was analyzed by unpaired two-tailed t-tests against the (b) control cells or (d) the 3250 cells (ns, not significant; **, p ≤ 0.01; ****, p ≤ 0.0001). (c,e) Numbers next to protein names indicate the molecular weight of the protein marker in kDa.

**
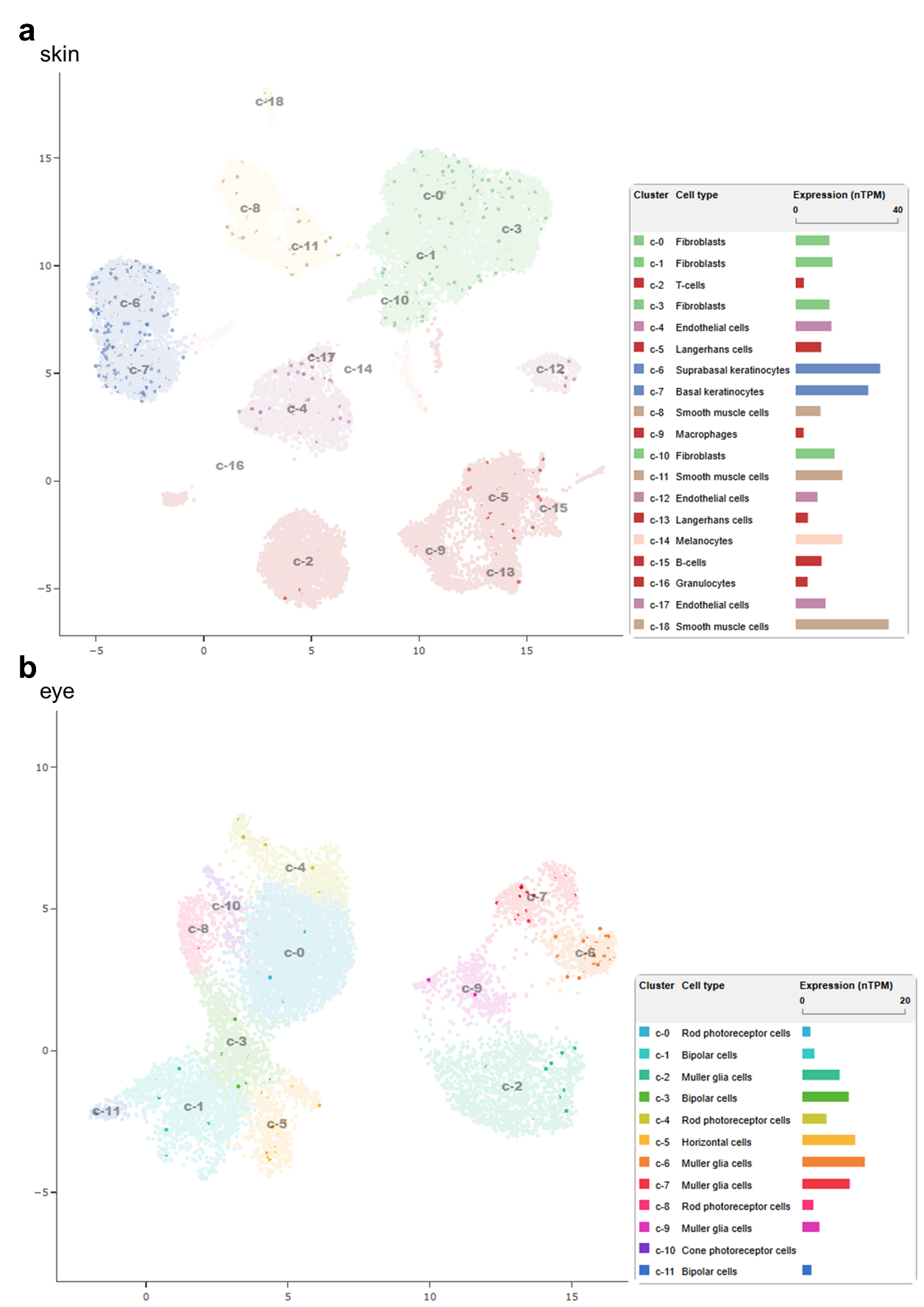
**

**
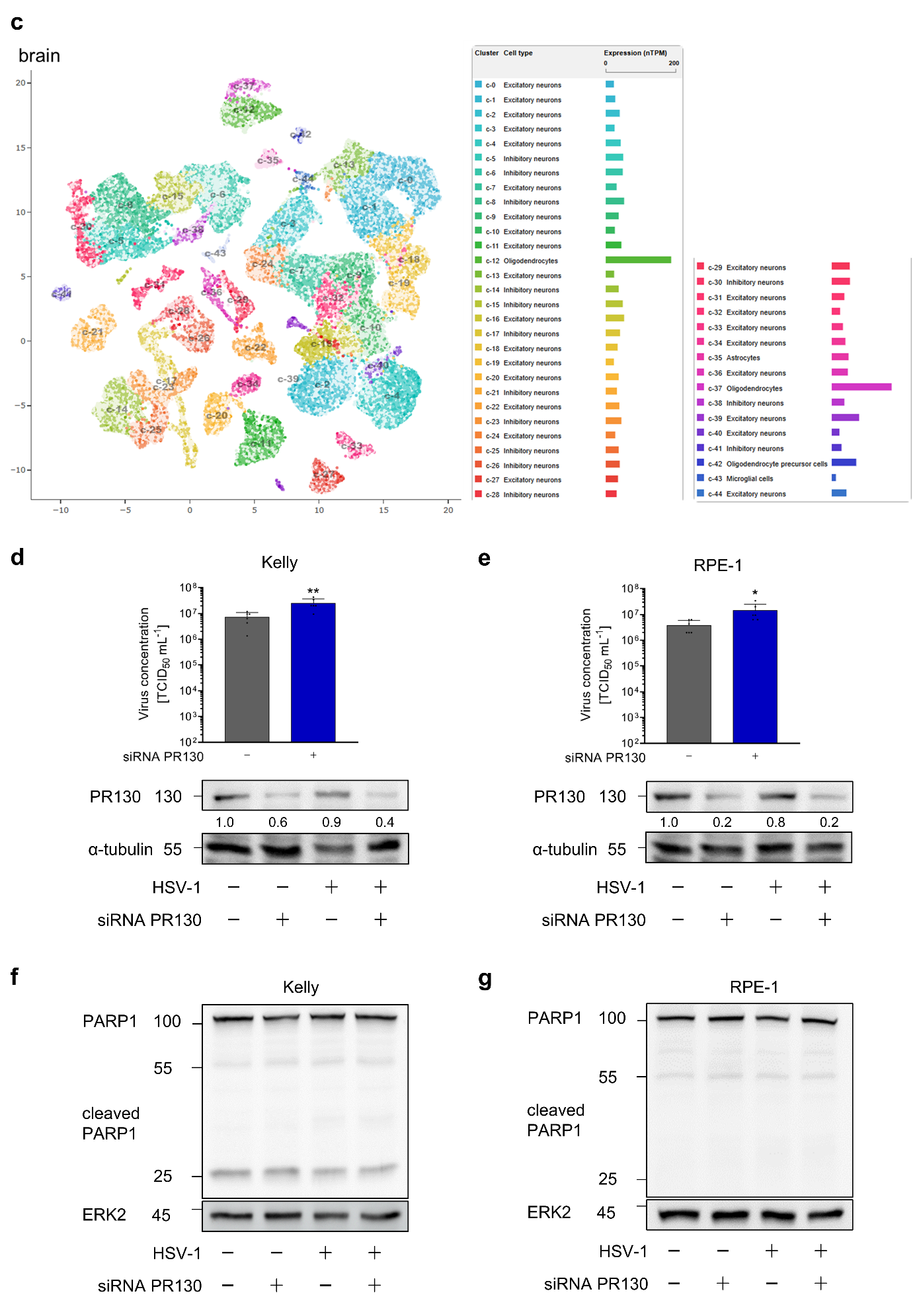
**

**Figure S3.** The Human Proteome Atlas indicates the presence of PR130 in (a) skin, (b) eye, and (c) brain tissues. (d) Kelly and (e) RPE-1 cells were transfected for 48 h with 100 pmol siRNA (siRNA #2), infected with HSV-1/GFP using a MOI of (d) 1 or (e) 0.1 and after a medium change were further incubated in the siRNA-containing medium. Virus concentrations were determined in the supernatants at 24 h p.i. Results of virus titrations are means (+SD) of TCID_50_ mL^-1^ values of 3 independent experiments, including 2 biological replicates. Statistical significance was analyzed by unpaired two-tailed t-tests against the control cells (*, p ≤ 0.05; **, p ≤ 0.01). Immunoblots were used to analyze the presence of PR130 and the loading control α-tubulin. Numbers next to protein names indicate the molecular weight of the protein marker in kDa. Numbers below represent the mean of 3 densitometric analyses relative to the loading control and normalized to the non-infected control cells (Figure S7p,q). (f) Kelly and (g) RPE-1 cells were transfected for 48 h with 100 pmol siRNA (siRNA #1), infected with HSV-1/GFP using a MOI of (f) 1 or (g) 0.1 and after a medium change were further incubated in the siRNA-containing medium. Immunoblots were used to analyze the levels of PARP1 and the loading control ERK2 at 24 h p.i. Numbers next to protein names indicate the molecular weight of the protein marker in kDa.

**
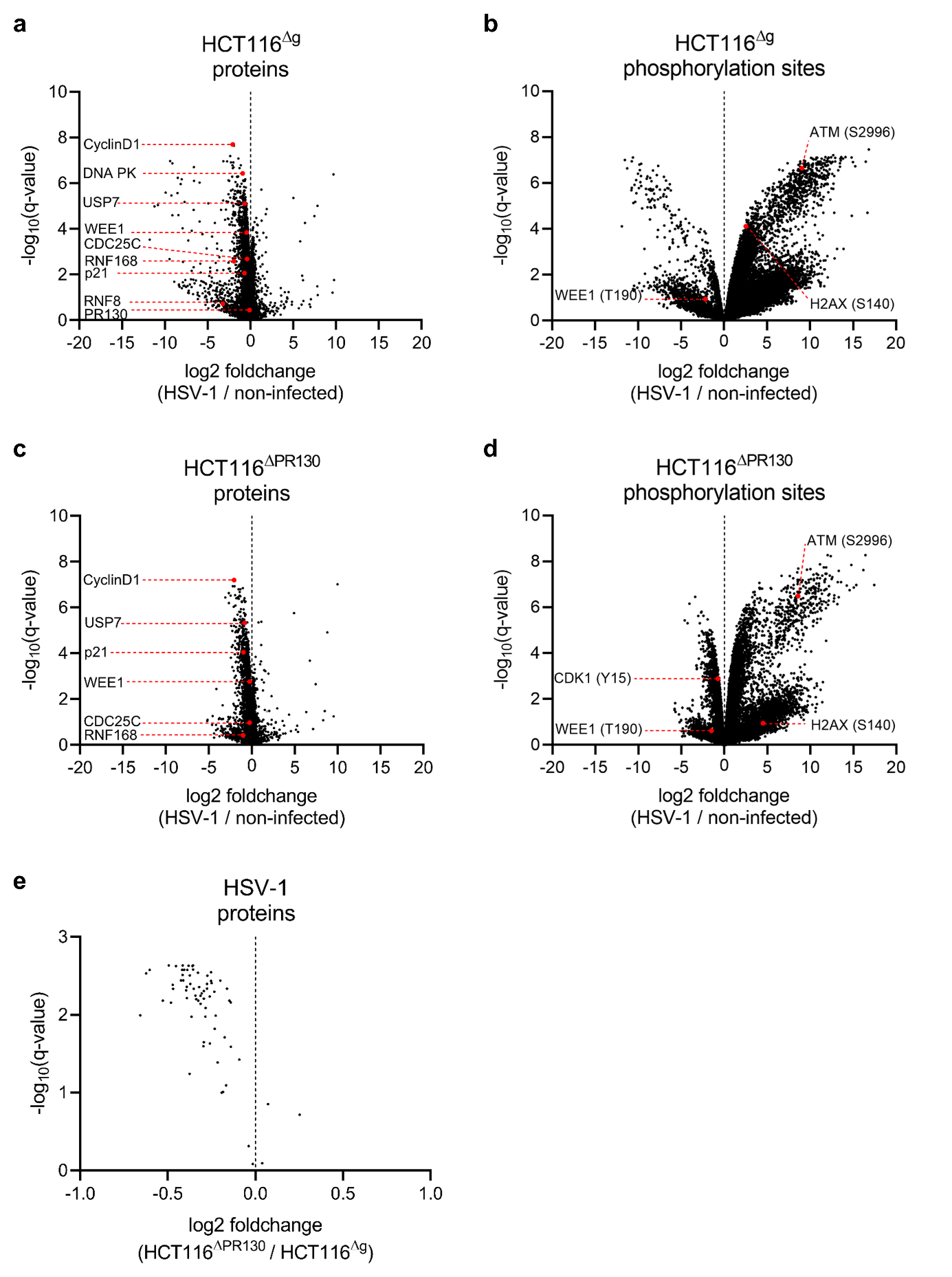
**

**Figure S4.** Mass spectrometry-based proteomics and phosphoproteomics reveal differentially expressed proteins and phosphorylation sites in PR130-deficient HCT116 cells. HCT116^Δg^ and HCT116^ΔPR130^ were infected with HSV-1/KOS using a MOI of 2. At 6 h p.i., cells were lysed in guanidinium chloride buffer and subjected to mass spectrometry. (a,b) Filtered host proteins. Values on the left: increase in non-infected HCT116^Δg^ cells and values on the right: increase in HSV-1-infected HCT116^Δg^ cells. (c,d) Filtered host proteins. Values on the left: increase in non-infected HCT116^ΔPR130^ cells and values on the right: increase in HSV-1-infected HCT116^ΔPR130^ cells. (e) Filtered HSV-1 proteins. Values on the left: increase in HCT116^Δg^ cells and values on the right: increase in HCT116^ΔPR130^ cells.

**
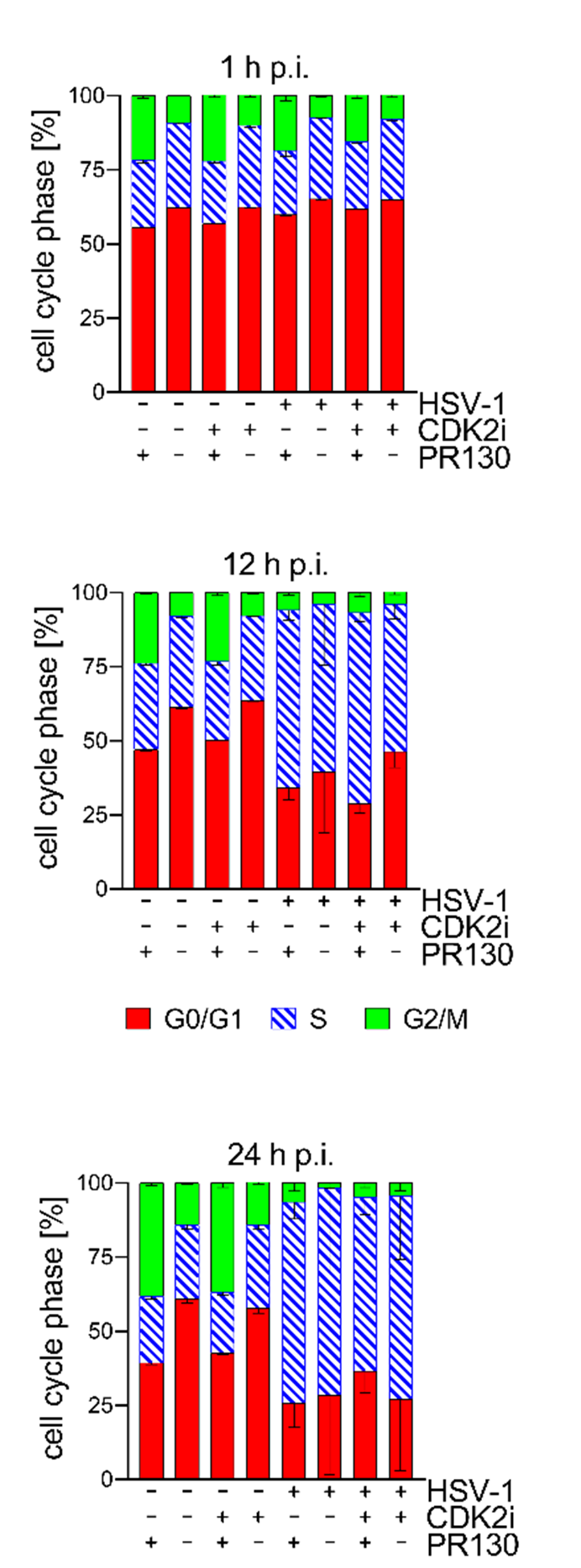
**

**Figure S5.** HCT116^Δg^ and HCT116^ΔPR130^ cells were treated with 5 µM CDK2i or DMSO as solvent control for 24 h and then infected with HSV-1/KOS using a MOI of 1. After medium change, cells were further incubated in CDK2i-containing medium for up to 24 h p.i. The cellular DNA content was determined at the indicated times by flow cytometry. Processed cells were labelled with PI (10 μg mL^-1^) to calculate different phases of cell cycle. The data were analyzed as means (- SD) of 3 independent experiments, including 2 biological replicates, and were obtained using ModFit LT 6.0 software.

**
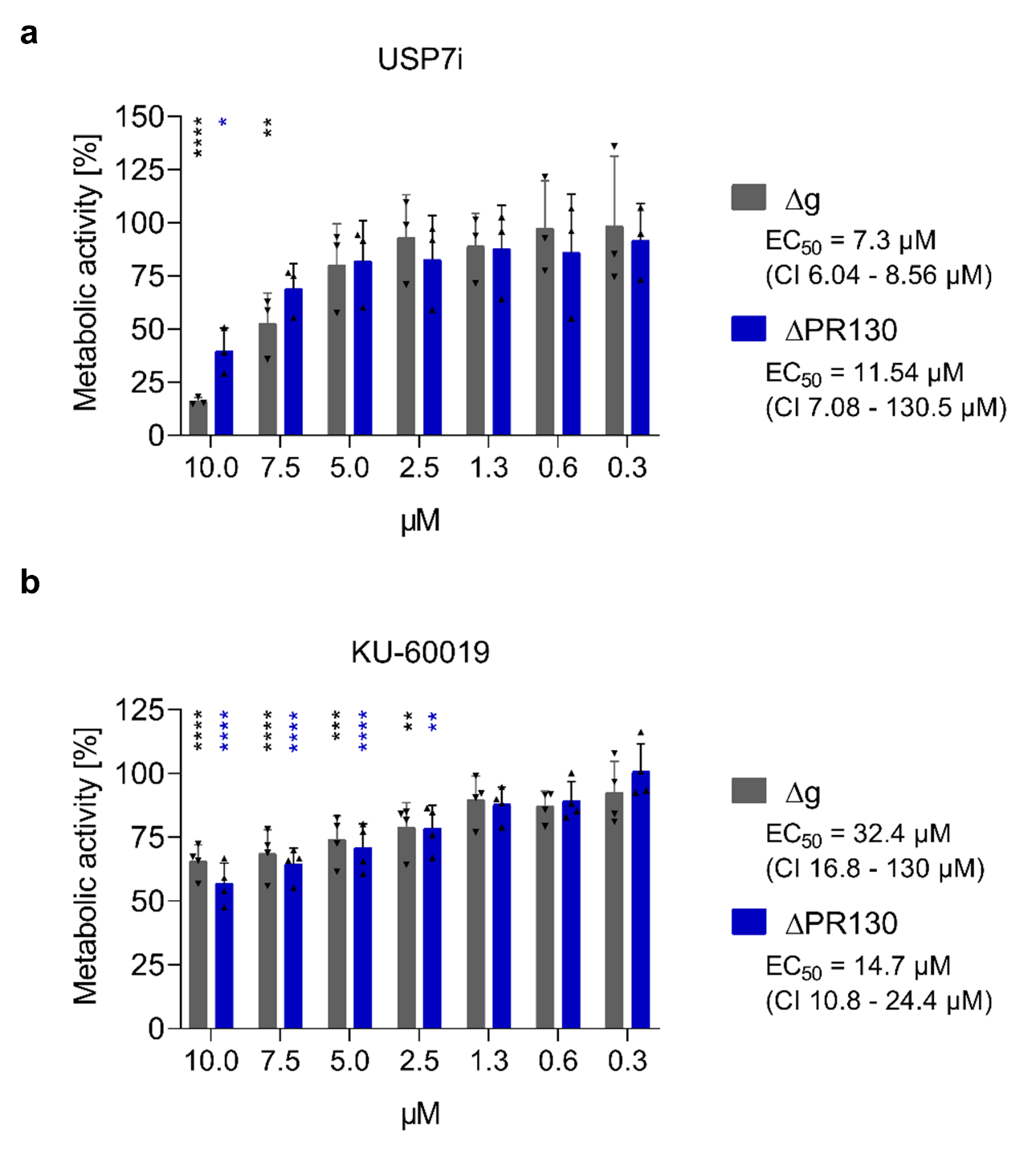
**

**Figure S6.** USP7i and KU-60019 are non-toxic in low-micromolar concentrations. HCT116^Δg^ and HCT116^ΔPR130^ cells were cultured in the presence or absence of (a) USP7i or (b) KU-60019 for 24 h. Metabolic activity was determined using MTT assays. Results were normalized to DMSO-treated control cells, which were arbitrarily set to 100%. Data are presented as the mean values (+SD) of 3 independent experiments, including 3 biological replicates. Statistical significance was analyzed by a two-way ANOVA with Dunnett`s multiple comparisons test to the DMSO-treated control cells (*, p ≤ 0.05; **, p ≤ 0.01; ***, p ≤ 0.001; ****, p ≤ 0.0001). Effective concentration 50% (EC_50_) values and 95% confidence intervals (CI) were determined by non-linear regression using GraphPad Prism software.

**
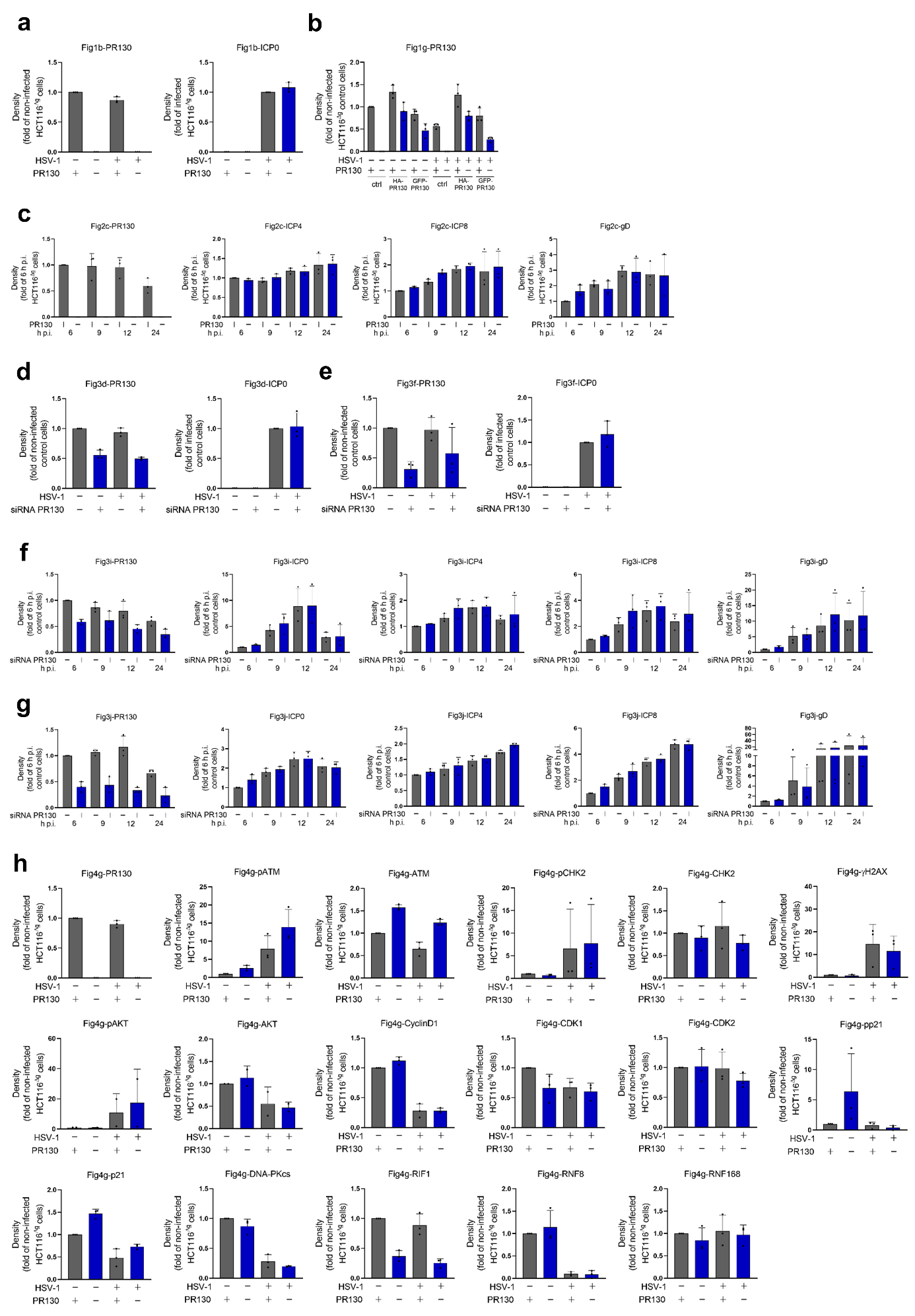
**

**
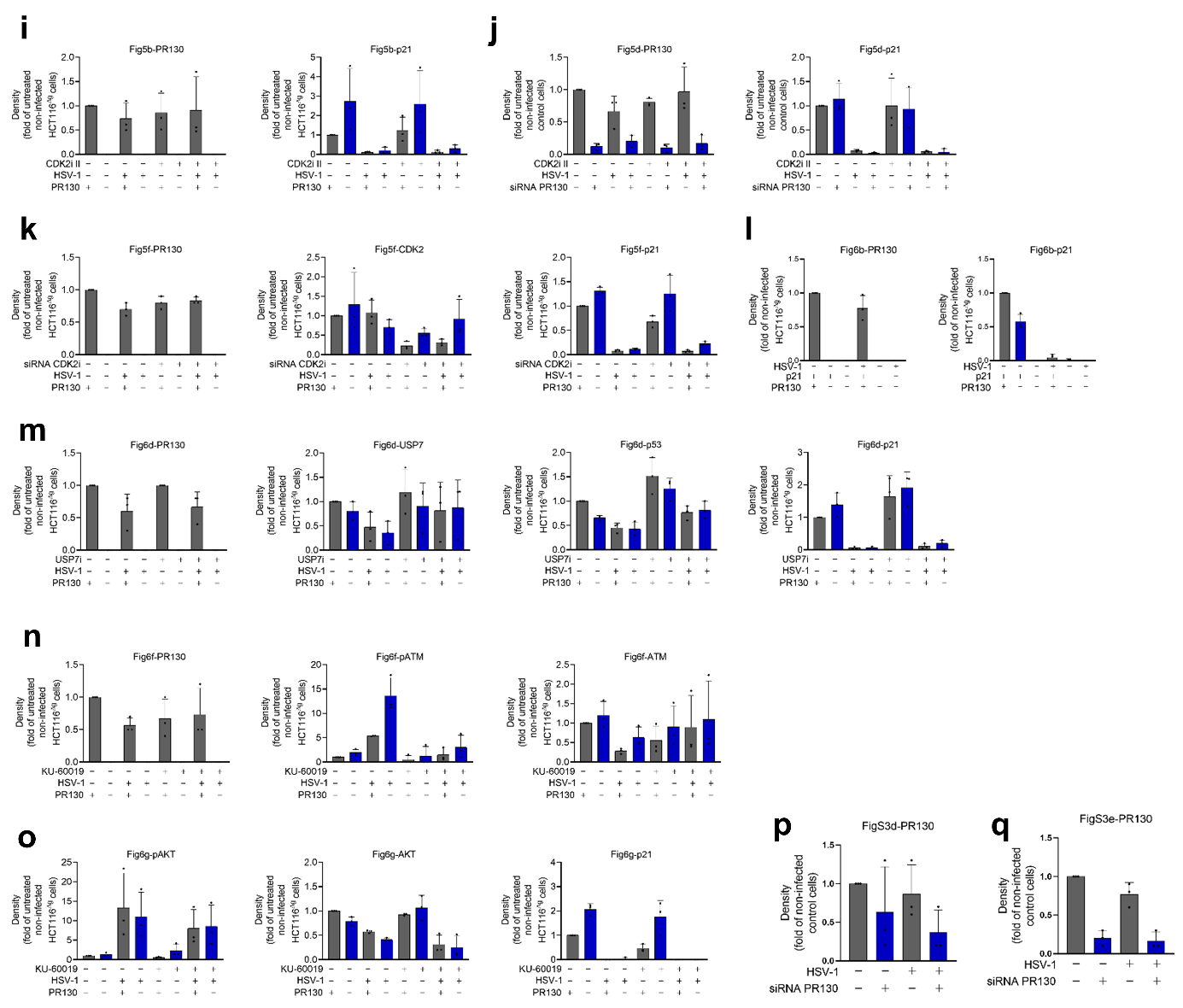
**

**Figure S7.** Densitometric analyses of 3 independent immunoblots of the respective figures. The values were normalized to the loading controls, further normalized to the respective non-infected or infected control cells and the mean (+SD) plotted.
